# Volatile composition and classification of *Lilium* flower aroma types and identification, polymorphisms, and alternative splicing of their monoterpene synthase genes

**DOI:** 10.1038/s41438-019-0192-9

**Published:** 2019-10-01

**Authors:** Fang Du, Ting Wang, Jun-miao Fan, Zhi-zhi Liu, Jia-xin Zong, Wei-xin Fan, Yuan-huai Han, Donald Grierson

**Affiliations:** 1 College of Horticulture, Shanxi Agricultural University, 030801 Taigu, Shanxi China; 2 College of Horticulture, Nanjing Agricultural University, 210095 Nangjing, Jiangsu China; 3 Experimental Teaching Center, Shanxi Agricultural University, 030801 Taigu, Shanxi China; 4 College of Agriculture, Shanxi Agricultural University, 030801 Taigu, Shanxi China; 5 Plant & Crop Sciences Division, School of Biosciences, University of Nottingham, Sutton Bonington Campus, Loughborough, LE12 5RD UK; 6 Department of Horticulture, College of Agriculture & Biotechnology, Zhejiang University, 310058 Hangzhou, China

**Keywords:** Secondary metabolism, Transcriptomics

## Abstract

Lily is a well-known ornamental plant with a diversity of fragrant types. Basic information on lily floral scent compounds has been obtained for only a few accessions, and little is known about *Lilium* aroma types, the terpene synthase genes that may play roles in the production of key volatiles, or the range of monoterpenes that these genes produce. In this study, 41 cultivars were analyzed for volatile emissions, and a total of 46 individual volatile compounds were identified, 16 for the first time in lilies. Lily accessions were classified into six groups according to the composition of major scent components: faint-scented, cool, fruity, musky, fruity-honey, and lily. Monoterpenes were one of the main groups of volatiles identified, and attention was focused on terpene synthase (*TPS*) genes, which encode enzymes that catalyze the last steps in monoterpene synthesis. Thirty-two candidate monoterpene synthase cDNAs were obtained from 66 lily cultivars, and 64 SNPs were identified. Two InDels were also shown to result from variable splicing, and sequence analysis suggested that different transcripts arose from the same gene. All identified nucleotide substitution sites were highly correlated with the amounts of myrcene emitted, and InDel site 230 was highly correlated with the emission of all major monoterpenoid components, especially (E)-*β*-ocimene. Heterologous expression of five cDNAs cloned from faint-scented and strong-scented lilies showed that their corresponding enzymes could convert geranyl diphosphate to (E)-*β*-ocimene, *α*-pinene, and limonene. The findings from this study provide a major resource for the assessment of lily scent volatiles and will be helpful in breeding of improved volatile components.

## Introduction

Floral scents have attracted the attention of people from ancient times; they are widely used in perfumes, food flavorings, and cosmetics, and in addition to flower color, they form the basis of important commercial traits of ornamental plants. However, fragrance has often been overlooked in breeding, and many fragrance traits have been lost over time due to artificial selection^1^. Detailed molecular genetic analysis can ameliorate some drawbacks of classical plant breeding^1^ and has been utilized in plants such as petunia and snapdragon, which have been used as models for investigating the synthesis and regulation of fragrance^2^. To facilitate the genetic improvement of fragrance, either by genetic engineering or marker-assisted breeding, basic qualitative and quantitative information on floral fragrance composition and quality and expression of related genes is required. Floral volatile profiling has been carried out with many scented flowering plants, and abundant volatiles have been identified, including terpenoids, phenylpropanoids/benzenoids, and fatty acids in *Osmanthus*^3^, *Dianthus*^4^, *Syringa*^5^, *Polianthes tuberosa*^6^, *Rosa*^7^, orchids^8^, freesia^9^, and *Pelargonium*^10^. There is great variation in odor strength, even within one species, and plants have been divided into groups according to the sensory characteristics of their fragrance. Roses were the first flowers to be classified based on their scent compounds. There are seven recognized rose fragrances: rose, nasturtium, orris, violet, apple, lemon, and clove, according to Le Grice^11,12^, which has led to the breeding of modern roses for fragrance and contributed to the appeal of rose scents in flower markets^13^. Similar classification of flower aromas has also been conducted in other ornamental plants to encourage additional efforts to breed fragrant plants to increase their market value. *Dianthus* fragrances were divided into four groups based on their scent characteristics, medicinal fragrance, citrus-like, green leafy odor, and nonscented^4^ and tulip cultivars into nine groups: anise, citrus, fruity, green, herbal, herbal-honey, rosy, spicy, and woody^13^.

Lily, one of the world’s best known ornamental plants, has a diversity of fragrance types, from weak scented to strong scented, which makes this flower a rich biochemical and genetic resource for aromas and flavors^14^. Nine different types of lilies have been classified by the Royal Horticultural Society according to parentage and flower shape: Asiatic hybrids (A); Martagon hybrids (M); European hybrids; American hybrids; Longiflorum hybrids (L); Trumpet and Aurelian hybrids (T); Oriental hybrids (O); interdivisional hybrids; and all species, their varieties, and forms^15^. With the development of intersectional hybridization techniques, new interdivisional hybrid lily cultivars have been introduced, including LA (Longiflorum × Asiatic), OT (Oriental × Trumpet), and LO (Longiflorum × Oriental)^16^. Over 60 volatile compounds have been identified from different lily cultivars^14,17^, and differences in the content and abundance of volatile molecules are believed to underlie the differences in lily fragrances^14,18^. Tepal tissues are major sources of floral scent compounds compared to the carpel and stamen^14,17^. Scent emissions occur in a circadian rhythm^17^ and function in nature to attract hawkmoths for pollination^19^. Lily volatiles that have been reported include terpenoids, benzenoids/phenylpropanoids, fatty acid derivatives, and nitrogen-containing and amino acid-derived compounds^14,17^. Compared with scented lilies, the release of monoterpene compounds by nonscented lilies is either very low or undetectable^17,18^. Although basic information on lily floral scent compounds and emission patterns has been obtained in recent years, little is known about the classification of aroma types of *Lilium*. Such information would be useful for understanding the fragrance character of lilies and could be utilized for the selection and breeding of new varieties.

In most cases, floral scents are mixtures of many compounds, but there are always major compounds that contribute most significantly to typical scents. Benzyl acetate is the characteristic floral scent compound in *Prunus mume*^20^, phenylacetaldehyde in petunia “TX-794”^21^, benzenoids in carnation^22^, and monoterpenes in *Lilium* “Siberia”^23^. Monoterpenes are a class of terpenes that consist of two isoprene units synthesized mainly through the 2-C-methyl-D-erythritol-4-phosphate (MEP) pathway in the plastid^24^. Monoterpene synthase genes (monoTPSs) are key genes that encode enzymes that catalyze the last steps in the MEP pathway, converting geranyl diphosphate (GPP) into a range of monoterpenes^24,25^ (Supplementary Fig. 1). To date, monoTPSs have been identified in many plants, including *Thymus albicans*^26^, *Camelina sativa*^27^, and *Alstroemeria*^28^, and have been well described in *Arabidopsis*^29,30^ and *Eucalyptus*^30,31^. *TPS* genes belong to a mid-sized gene family and range in number from 1 in *Physcomitrella patens* to 113 in *Eucalyptus grandis*^30,32,33^. An important characteristic of *TPS* enzymes is that they yield multiple products, depending on the substrate^32,34^. Steele et al.^35^ reported 52 different terpenes produced by one enzyme in *Abies grandis*, and at least 40 multisubstrate *TPS*s have been identified^36^.

Recently, two *LiTPS* genes have been cloned, one from *Lilium* “Siberia” (NCBI accession number KF734591), consisting of a 1761-bp open reading frame (ORF) and encoding a 587 amino acid protein^37^, and another, *LhTPS* cloned from “Belladonna” (NCBI accession number KR998333), with an ORF of 1758 bp corresponding to 586 amino acids^14^. However, no information is available on the genetic diversity of monoTPSs or their relationship to floral scent in lily. The objectives of this study were to (1) characterize floral volatile emissions in 41 lily accessions with different aroma types and produce a basic classification of aroma types in *Lilium*, (2) identify the nucleotide and haplotype diversity (Hd) for *TPS*s in a panel of 66 lily species/cultivars and evaluate its use for functional characterization, and (3) characterize the *TPS* gene transcripts from different lily accessions.

## Results

### Analysis and identification of novel floral scents from new *Lilium* hybrids and cultivars

Previous studies of lily fragrances have compared faint-scented or strong-scented flowers from a few different lily types, including OT hybrids “Belladonna”, “Conca D’ Or”, “Robina”, and “Yelloween”; Oriental hybrids “Marco Polo”, “Siberia”, “Sorbonne”, “Sunshine Borland”, “Love story”, “Pink Champion”, “Santander”, and “Tarrango”; Longiflorum hybrid “White Heaven”; Asiatic hybrids “Tresor”, “White Wizard”, and “Red Wizard”; and LA hybrids “Music” and “Ceb Dazzle”^14,17,18^. In recent years, Trumpet hybrids, LO hybrids, and other new cultivars with distinct fragrances have become available. In this study, 41 cultivars of 12 different types (Table 1) were analyzed for volatile emissions, including 38 accessions that have not been investigated previously. A total of 46 individual volatile compounds were identified, ranging from 8 volatile components for “Pearl Melanie” to 34 components for “Regale album” (Supplementary Table 1). Of those components, 16 were identified for the first time in lilies, while the remainder had been identified in previous studies. Most newly identified compounds were either sesquiterpenes or derived from fatty acids (Supplementary Table 1).

**Table 1:** Lily accessions used in this study

No.	Type	Accession	No.	Type	Accession	No.	Type	Accession	No.	Type	Accession
1	W	*L. davidii* var. *unicolor* Pinglu^b^	22	A	*L. tigrinum Splendens* ^b^	43	OT	Friso^b^	64	LA	Golden Stone^a,b^
2	W	*L. davidii* var. *unicolor*^b^	23	A	Discoteca^b^	44	OT	Urandi^b^	65	LA	Brindisi^a,b^
3	A	Easy Waltz^b^	24	A	Levi^a,b^	45	OT	Conca D’ Or^a,b^	66	LA	Pavia^a^
4	A	Little Kiss^b^	25	A	Elodie^a,b^	46	OT	Robina^b^	67	LA	Surrender^a,b^
5	A	Easy life^b^	26	A	Rosellas Dream^a,b^	47	OT	Olympic Torch^b^	68	LO	Triumphator^a,b^
6	A	Peach Dwarf^b^	27	A	Matrix^b^	48	OT	Yelloween^b^	69	LO	White Triumphator^a^
7	A	Red Twin^b^	28	A	Sunset Matrix^b^	49	OT	Amarossi^b^	70	LO	Bell Song^a^
8	A	Ivory Pixie^b^	29	A	Golden Matrix^b^	50	OT	Outback^b^	71	LO	Pink Brilliant^a^
9	A	Abbeville Pride^b^	30	T	Pink Perfection^a,b^	51	OT	Forever^b^	72	LO	Pink Heaven^a^
10	A	Tailor Made^b^	31	T	Orange Planet^a,b^	52	OT	Pink Mist^a,b^	73	F	Julius^b^
11	A	Twosome^b^	32	T	Golden splendor^a^	53	OT	Garden Affair^a^	74	L	White Heaven^a^
12	A	Pink Pixie^b^	33	T	Regale Album^a^	54	OT	Palazzo^a^	75	O	Mona Lisa^b^
13	A	Navona^b^	34	T	Regale^a^	55	OT	Manissa^a^	76	O	Reeleeze^b^
14	A	Tiny Ghost^b^	35	AT	Pearl Melanie^a,b^	56	OT	Miss Feya^a^	77	O	Entertainer^b^
15	A	Tiny Invader^b^	36	AT	Pearl Loraine^a,b^	57	TO	Mister Right^a,b^	78	O	After Eight^b^
16	A	Tiny Pearl^b^	37	AT	Pearl Jessica^a,b^	58	TO	Mister Sandman^b^	79	O	Sorbonne^a,b^
17	A	Black Eye^a,b^	38	AT	Red Velvet^a,b^	59	TO	Leslie Woodriff^b^	80	O	Siberia^b^
18	A	Pink Blossom^b^	39	AT	Pink Flavour^a,b^	60	TO	Mister Cass^a,b^	81	O	White Dream^b^
19	A	Annemaries Dream^b^	40	AT	Red Life^b^	61	TO	Beverly Dreams^ab^	82	O	Brasilia^a^
20	A	Vermeer^b^	41	TA	Night Rider^a^	62	TO	Robert Swanson^a^	83	LP	Fusion^a^
21	A	Tiny Diamond^a,b^	42	OT	Zambesi^b^	63	LA	Bright Diamond^a,b^	84	AL	Lady Alice^a^

*W* wild species, *A* Asiatic hybrids, *T* Trumpet hybrids, *AT* Asiatic × Trumpet hybrids, *TA* Trumpet × Asiatic hybrids, *OT* Oriental × Trumpet hybrids, *TO* Trumpet × Oriental hybrids, *LA* Longiflorum × Asiatic hybrids, *LO* Longiflorum × Oriental hybrid, *O* Oriental hybrids, *F* *L. formolongi*, *L* Longiflorum, *LP* *L. longiflorum* *×* *L. pardalinum*, *AL* Aurelian hybrids × *Lilium henryi*

^a^Accessions used for floral scent analysis

^b^Cultivars used for total RNA extraction

### Grouping of lily cultivars based on their fragrance

Each cultivar has a particular floral scent profile. There are 16 scent compounds that are considered major scent components and whose relative amounts are >10% in some accessions, including 5 monoterpenoids ((E)-*β*-ocimene, myrcene, *α*-pinene, eucalyptol, and linalool), 1 sesquiterpene (caryophyllene), 3 benzenoids (methyl benzoate, ethyl benzoate, and toluene), 1 phenylpropanoid-related (naphthalene), and 6 fatty acid-derived compounds (3-hexen-1-ol, methyl 2-methylbutyrate, methyl tiglate, methyl hexanoate, methyl octanoate, and 2-ethenyl-1,1-dimethyl-3-methylenecyclohexane) (Supplementary Table 1). Methyl benzoate was the only component detected in all accessions. (E)-*β*-ocimene, naphthalene, and methyl 2-methylbutyrate were detected in 39 accessions. Myrcene was also widely detected in 36 accessions (Supplementary Table 1).

Major scent compounds were classified into five groups (herbal, fruity, cool, floral, and spicy) based on their odor descriptions (Supplementary Table 2), and the scents of cultivars were classified into six groups based on the ratios of total amounts of scent compounds for each group (Table 2).

**Table 2:** Classification of lily cultivars by their relative proportions of different scent compounds

Group	Cultivars	Major scent compounds (%)				
		Herbal	Fruity	Cool	Floral	Spicy
Faint scented	Black Eye	7.27	11.03	53.80	5.04	0.13
	Tiny Diamond	13.3	33.77	6.39	23.22	0.3
	Levi	12.61	52.39	12.15	–	1.11
	Elodie	4.97	40.11	25.29	–	4.36
	Rosella’s Dream	6.93	22.51	5.23	10.77	22.27
	Pearl Melanie	–	33.06	58.45	–	–
	Pearl Loraine	12.04	43.05	15.95	5.79	5.43
	Pearl Jessica	14.54	22.70	12.89	–	26.45
	Red Velvet	1.37	58.57	12.06	–	–
	Lady Alice	6.02	52.00	5.91	–	14.58
	Pink Flavour	28.08	33.86	12.71	–	–
	Surrender	37.28	31.65	0.27	13.78	1.96
	Fusion	39.81	34.98	1.72	3.39	–
	Bright Diamond	26.04	57.40	0.74	6.03	0.03
Cool	Robert Swanson	9.36	1.49	66.30	1.77	0.18
	Beverly Dreams	9.83	6.87	55.13	–	2.85
	Mister Right	11.71	8.89	41.88	–	0.34
	Mister Cass	16.30	4.86	57.22	4.60	0.23
Fruity scent	White heaven	6.11	65.61	0.47	16.12	–
	Golden Stone	4.47	69.35	0.22	13.38	–
	Brindisi	3.21	87.69	2.21	1.72	0.33
	Pavia	3.74	57.63	0.55	33.67	0.56
Musky scent	Pink Perfection	4.38	68.71	21.67	–	–
	Regale	5.87	34.72	20.94	2.33	–
	Golden Splendor	6.14	60.15	29.8	–	–
	Night Rider	6.95	44.75	31.93	–	2.32
	Regale Album	7.64	40.40	22.83	1.18	0.02
	Manissa	15.89	34.21	13.04	23.32	–
	Orange Planet	20.45	9.71	60.56	–	–
Fruity-honey scent	White Triumphator	1.38	81.75	0.12	–	–
	Garden Affair	4.28	52.30	14.75	–	–
	Triumphator	5.21	76.60	0.25	2.82	–
	Bell Song	10.96	48.53	0.15	23.77	–
	Pink Heaven	11.07	43.79	0.06	35.41	–
	Pink Brilliant	14.64	20.37	1.82	59.28	–
	Conca D’ Or	11.24	63.77	6.12	9.93	–
	Pink Mist	13.65	62.43	14.19	–	–
	Palazzo	16.54	46.90	18.37	12.22	–
Lily scent	Miss Feya	23.63	51.15	0.50	9.20	0.12
	Sorbonne	33.42	36.78	–	4.43	–
	Brasilia	34.72	45.00	0.19	6.43	0.01

#### **Group 1**, Faint scented

Group 1, which had hardly any scent, comprised 14 cultivars, mainly belonging to Asiatic hybrids and AT hybrids. Although some important scent compounds such as linalool and methyl benzoate were detected in some cultivars (e.g., “Tiny Diamond”, “Levi”, “Rossella’s Dream”, and “Pearl Loraine”), most humans are unable to detect any fragrance from these flowers by nose, although a few subjects in this study were able to detect a faint fragrance from flowers of “Pink Flavour”, “Lady Alice”, “Surrender”, “Fusion”, and “Bright Diamond”, which had relatively high levels of methyl benzoate and (E)-*β*-ocimene.

#### **Group 2**, Cool

Group 2 had a weak scent with a cooling nuance and contained four cultivars, “Robert Swanson”, “Beverly Dreams”, “Mister Right”, and “Mister Cass”. All of these cultivars, which are TO hybrids, emitted a high percentage of eucalyptol and exhibited notable morphological characteristics, with dark green leaves and crooked stems, especially in young plants.

#### **Group 3**, Fruity

Group 3, consisting of “White Heaven”, “Golden Stone”, “Brindisi”, and “Pavia”, had a light fresh fruit odor, and methyl benzoate was the major scent compound. “White Heaven” is a Longiflorum hybrid, and the other three cultivars are LA hybrids. Compared to the other cultivars, “Pavia” had a high level of linalool in addition to methyl benzoate, which produced a fresh fruit scent with a floral note.

#### **Group 4**, Musky

Group 4 comprised of seven cultivars, five Trumpet hybrids (“Pink Perfection”, “Orange Planet”, “Golden Splendor”, “Regale Album”, “Regale”), one TA hybrid (“Night Rider”), and one OT hybrid (“Manissa”). All of those cultivars had a musky odor derived from high levels of eucalyptol and methyl benzoate, which many people find distasteful, especially in a confined space.

#### **Group 5**, Fruity-honey

Nine cultivars belonging to the OT and LO hybrids had fruity-honey odors with relatively high levels of methyl benzoate. “Triumphator” and “White Triumphator” had relatively high levels of methyl 2-methylbutyrate in addition to high levels of methyl benzoate, contributing a fruity and sweet odor. “Bell Song”, “Pink Brilliant”, and “Pink Heaven” had methyl benzoate and linalool as the major scent compounds, in addition to (E)-*β*-ocimene and myrcene. Linalool has a typical pleasant floral odor, (E)-*β*-ocimene and myrcene (ocimene-type) have herbaceous odors, and the three cultivars thus smelled fruity and sweet with a floral note. “Conca D’ Or”, “Pink Mist”, “Garden Affair”, and “Palazzo” emitted methyl benzoate in addition to eucalyptol and ocimene-type as the major scent compounds, generating a fruity odor with a sweet resinous nuance.

#### **Group 6**, Lily

Group 6 contained “Miss Feya” (OT hybrid) and “Sorbonne” and “Brasilla” (Oriental hybrids), which had high levels of ocimene-type and methyl benzoate, producing a strong fragrance with a cananga odor. This scent is very popular and was designated “lily”, being so reminiscent of odors from cut Oriental hybrid lily flowers.

### Sequence alignment of *TPS* genes from different lily cultivars

Methyl benzoate might be important for the formation of lily fragrance since it was detected in all lily accessions. However, monoterpenes were predicted to be the main contributors to the difference in floral scent between scented and faint-scented lilies^23^. As shown in Supplementary Table 1, monoterpenes occur frequently in lilies but in different relative amounts. They are also present in some cultivars that are normally considered faint scented. Monoterpenes are known to be synthesized mainly by the MEP pathway^36^, and monoTPSs encode the enzymes catalyzing the final steps of the MEP pathway. We tested the hypothesis that it was possible to identify polymorphic sites in lily flower-expressed monoTPSs related to the production of monoterpenes.

**Fig. 1:**
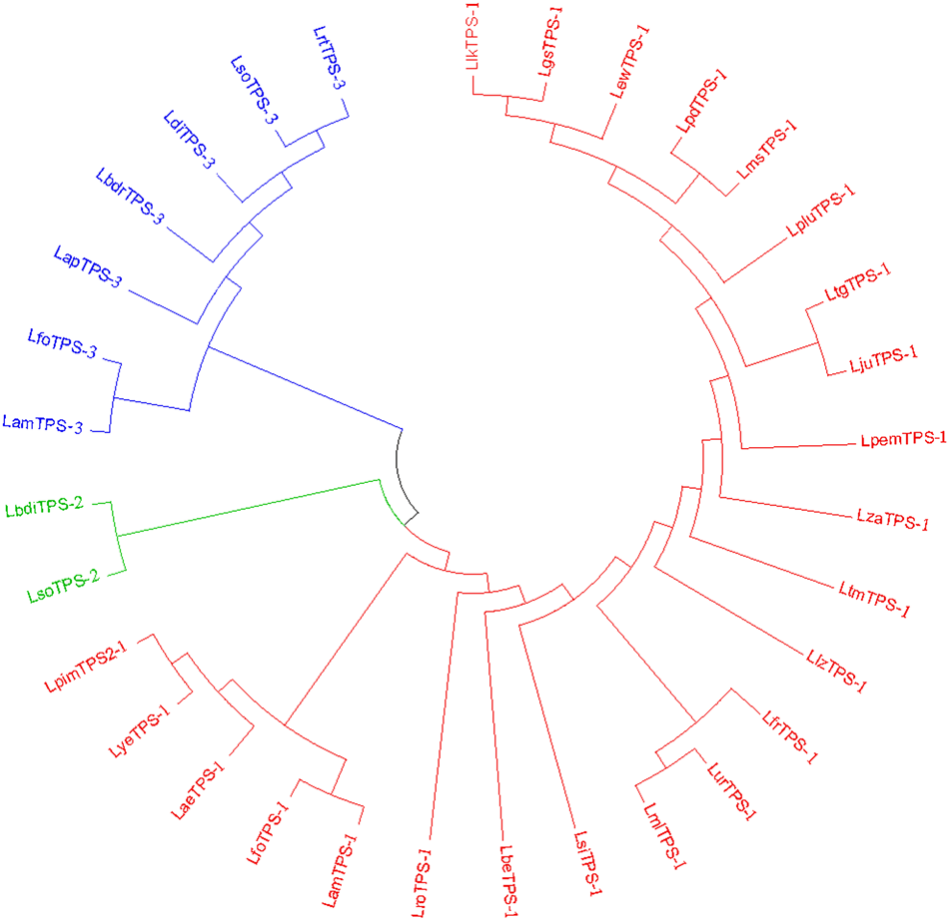
Unrooted phylogenetic tree of 32 putative lily TPS cDNAs analyzed using the neighbor-joining method of MEGA 6. Members of LTPS-1 are highlighted in red, LTPS-2 in cyan, and LTPS-3 in navy blue

Thirty-two monoTPS cDNAs with high similarity were identified and sequenced from 66 lily accessions, and the sequences were submitted to NCBI (Table 3). The cDNAs were divided into three groups: LTPS-1, LTPS-2, and LTPS-3 (Fig. 1). LTPS-1 included the largest number of cDNAs (23 cDNAs in total) characterized by an ORF of 1761 bp encoding a putative LTPS protein of 586 amino acids. LTPS-3 included 7 cDNAs, with 97.71% homology to the LTPS-1 group, characterized by an ORF of 1647 bp encoding a putative LTPS protein of 548 amino acids. There were only two cDNAs belonging to LTPS-2, with 98.59% and 94.44% homology to LTPS-1 and LTPS-3, respectively. The ORF of LTPS-2 is 1773 bp and encodes a putative LTPS protein of 590 amino acids.

**Table 3:** Distribution of LTPS-1, LTPS-2, and LTPS-3 in 66 lily cultivars

Group	Material	Gene name	Accession no.
LTPS-1	Little Kiss, Pink Pixie, Vermeer, Levi, Rosella’s Dream, Sunset Matrix, Pearl Loraine, Surrender, Sorbonne^a^, Easy Life^b^, Ivory Pixie, Abbeville Pride, Navona, Tiny Invader, Tiny Pearl, Pink Blossom, Tiny Diamond, *L. tigrinum* Splendens, Matrix, Pearl Jessica, Red Life^b^, Mister Right, Leslie Woodriff, Mister Cass, Pink Mist^c^, Entertainerm, Reeleeze^b^	*LlkTPS-1*	MH203231
	Easy Waltz, Red Velvet, Pink Perfection^b^	*LewTPS-1*	MH203230
	Tailor Made	*LtmTPS-1*	MH203236
	Tiny Ghost	*LtgTPS-1*	MH203239
	*L. davidii* var. *unicolor* “Pinglu”	*LpluTPS-1*	MH203273
	*Lilium davidii* var. *unicolor*	*LlzTPS-1*	MH203274
	Pearl Melanie	*LpemTPS-1*	MH203268
	Mona Lisa	*LmlTPS-1*	MH203251
	Olympic Torch, Yelloween, Outback	*LyeTPS-1*	MH203282
	Pink Mist^c^	*LpimTPS2-1*	MH203288
	After Eight	*LaeTPS-1*	MH203279
	Peach Dwarf	*LpdTPS-1*	MH203233
	Black Eye	*LbeTPS-1*	MH203242
	Zambesi	*LzaTPS-1*	MH203255
	Friso	*LfrTPS-1*	MH203256
	Urandi	*LurTPS-1*	MH203257
	Robina	*LroTPS-1*	MH203258
	Mister Sandman	*LmsTPS-1*	MH203265
	Golden Stone	*LgsTPS-1*	MH203261
	Amarossi^b^	*LamTPS-1*	MH203283
	Forever^b^	*LfoTPS-1*	MH203287
	Julius^d^	*LjuTPS-1*	MH203263
	Siberia	*LsiTPS-1*	MH203254
LTPS-2	Sorbonne^a^, Julius^d^, Conca D’ Or, Orange Planet, Annemarie Dream	*LsoTPS-2*	MH203303
	Bright Diamond	*LbdiTPS-2*	MH203304
LTPS-3	Easy life^b^, Discoteca, Elodie, Pink Flavour, Red Life^b^, Pink Perfection^b^, Triumphator, White Dream	*LdiTPS-3*	MH203298
	Sorbonne^a^, Reeleeze^b^, Brindisi, Golden Matrix, Twosome	*LsoTPS-3*	MH203275
	Red Twin	*LrtTPS-3*	MH203295
	Abbeville Pride	*LapTPS-3*	MH203296
	Amarossi^b^	*LamTPS-3*	MH203284
	Forever^b^	*LfoTPS-3*	MH203286
	Beverly Dreams	*LbdrTPS-3*	MH203280

^a^Cultivars in which three types of LTPS were cloned

^b^Cultivars in which both LTPS-1 and LTPS-3 were cloned

^c^Cultivars in which two LTPS-1 were cloned

^d^Cultivars in which both LTPS-1 and LTPS-2 were cloned

Table 3 shows the distribution of three types of terpene synthase transcripts, LTPS-1, LTPS-2, and LTPS-2, found in different lily cultivars examined in this study. LTPS-1 was found in 52 (78.8%) lily cultivars. LTPS-3 was cloned from 18 cultivars, accounting for 27.3%, and 6 (9.1%) cultivars contained LTPS-2. “Sorbonne” was the only cultivar from which three types of LTPS were cloned. “Julius” was the only cultivar from which both LTPS-1 and LTPS-2 were cloned. LTPS-1 and LTPS-3 were cloned from seven cultivars, “Easy Life”, “Abbeville Pride”, “Red Life”, “Reeleeze”, “Pink Perfection”, “Amarossi”, and “Forever”. Ten cultivars had only LTPS-3, and four cultivars (“Annemarie’s Dream” “Orange Planet” “Conca D’ Or” and “Bright Diamond”) had only LTPS-2.

### Nucleotide diversity of LTPS-1, LTPS-2, LTPS-3, and haplotypes

Sequence alignment of the 32 monoTPS sequences from lily showed 2 InDel polymorphisms (230, 873) and 64 single-nucleotide polymorphisms (SNPs). Compared to LTPS-1, 12 base pairs were inserted in LTPS-2 (230), and 114 base pairs were deleted in LTPS-3 (873). Of the 64 SNPs, 25 were synonymous mutations, while the other 39 altered the amino acid sequences (Supplementary Table 3).

Fifty-five, 8, and 41 SNP sites were identified for LTPS-1, LTPS-2, and LTPS-3, respectively, of which 33, 6, and 17 were nonsynonymous substitutions. Nucleotide diversity (*π*) was determined for LTPS-1, LTPS-2, and LTPS-3 ORFs using the SNPs identified in the lily accessions. Overall, nucleotide diversity was higher for LTPS-1 (*π* = 0.010) than for LTPS-3 (*π* = 0.006) and LTPS-2 (*π* = 0.001). Hd was approximately 1.0 for the three LTPS groups (Table 4).

**Table 4:** Nucleotide diversity of LTPS-1, LTPS-2, and LTPS-3 in the lily accessions

Group	SNP site	Nonsynonymous substitution	Nucleotide diversity (*π*)	No. of haplotypes	Haplotype diversity (Hd)
LTPS-1	55	33	0.010	23	0.99
LTPS-2	8	6	0.001	2	1.00
LTPS-3	41	17	0.006	7	1.00

### Comparison of lily genomic DNA sequences and LTPS flower transcripts

**Fig. 2:**
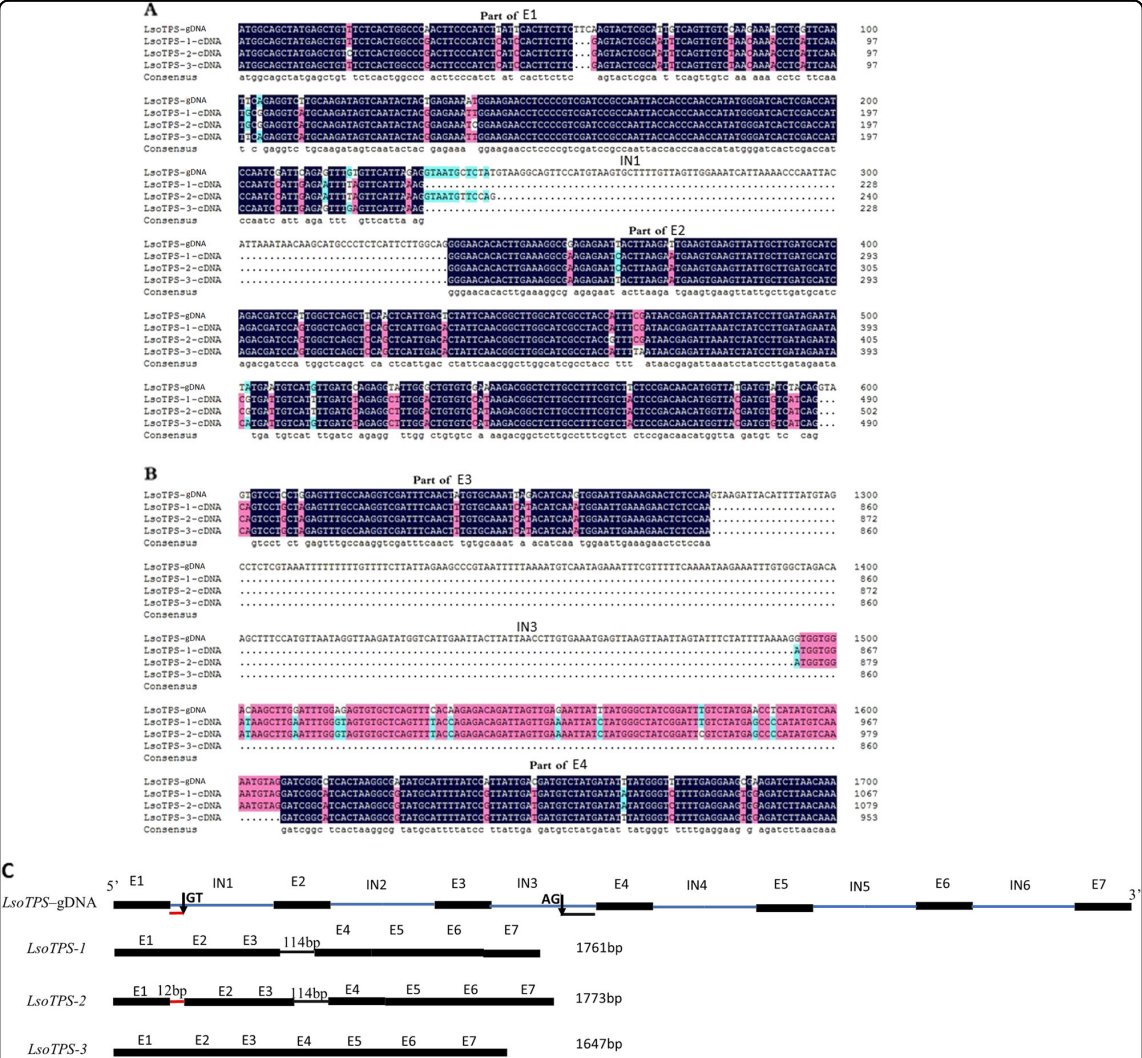
Alignment of the Lily “Sorbonne” genomic DNA sequence (*LsoTPS* gDNA) with three cDNA sequences (*LsoTPS-1*, *LsoTPS-2*, and *LsoTPS-3*). **a** From the first exon to the second exon. **b** From the third exon to the fourth exon. Navy blue denotes identical bases among all sequences, pink denotes identity among three out of four sequences, and cyan denotes identity among half of the sequences. Missing bases are indicated with dashes. **c** Diagram depicting the splice variants of *Lilium* monoterpene synthase genes. e exon, IN intron. E1–E7 and IN1–IN6 represent the seven exons and six introns, respectively, with exons shown as black boxes and introns as blue lines. Vertical arrows indicate the alternative splicing sites with the splice sequence indicated. The red line denotes the 12 bp 5′ splicing fragment from intron 1, and the dark gray line denotes the 114 bp 3′ splicing fragment of intron 3

The cultivar “Sorbonne” was the only accession from which three types of LTPS were cloned, and it was chosen for DNA cloning to investigate the InDel relationships between LTPS-2 and LTPS-3 at the DNA level. A total length of 2661 bp genomic LTPS DNA was cloned from “Sorbonne” (*LsoTPS*–gDNA, NCBI accession number MH618207) and aligned with the cDNAs of *LsoTPS-1* (MH203231), *LsoTPS-2* (MH203303), and *LsoTPS-3* (MH203275) by the DNAMAN software. Seven exons and six introns were found for the LTPS genomic DNA. As shown in Fig. 2, the additional 12 bases of *LsoTPS-2*, which are not present in *LsoTPS-1* and *LsoTPS-3*, are not actually due to an insertion in the DNA-coding sequence but rather arise from the use of an alternative 5′ splice site in the first intron. Furthermore, the 114 bases missing from *LsoTPS-3* compared to *LsoTPS-1* and *LsoTPS-2* are not due to the deletion of any DNA sequence but rather arise from the use of an alternative 3′ splice site in the third intron. This suggests that the explanation for the presence of the *LTPS-1*, *LTPS-2*, and *LTPS-3* transcripts might be variable splicing of transcripts from an *LTPS* gene.

### Correlation of scent compounds emitted and *TPS* gene nucleotide substitutions

**Fig. 3:**
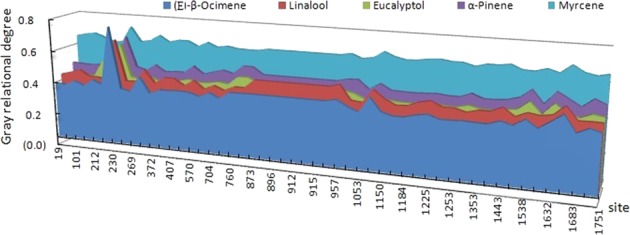
Gray relational analysis between monoterpene volatile compounds and *TPS* nucleotide substitutions using the DPS software

Fifty-one SNPs were identified among the cDNA sequences from the 23 cultivars from which floral scent components were identified. Gray relational analysis was used to evaluate the gray relational degree (GRD) between the major monoterpene volatile compounds emitted and the 51 *TPS* nucleotide substitutions^38,39^ using the DPS V12.01 software. The compound that had the highest correlation with all nucleotide substitution sites (GRD > 0.57) was myrcene (Fig. 3). The substitution site 230–241, which corresponds to the insertion of 12 bp, was highly correlated with all major monoterpene volatile compounds (GRD > 0.59), especially (E)-*β*-ocimene (GRD > 0.72) (Fig. 3).

### Characterization of LTPS-1, LTPS-2, and LTPS-3

**Fig. 4:**
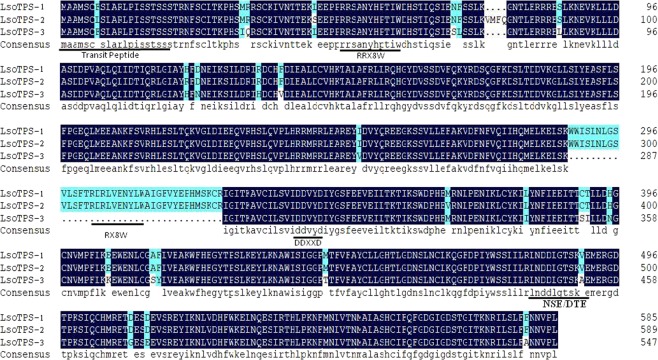
Alignment of the deduced amino acid sequences of LsoTPS-1, LsoTPS-2, and LsoTPS-3. The N-terminal transit peptide and conserved amino acid motifs RRX8W, RX8W (missing in LsoTPS-3 due to the alternative splicing), DDXXD, and NSE/DTE are indicated. RRX8W has been suggested to facilitate the isomerization–cyclization reaction^10^. DDXXD and NSE/DTE are speculated to form the binding site for the substrate (GPP)–divalent metal cation (Mg^2+^, Mn^2+^, etc.)^32^. RX8W is speculated to be the substrate-binding pocket according to an NCBI CD search (https://www.ncbi.nlm.nih.gov/Structure/cdd/wrpsb.cgi?tdsourcetag)

The translation of all *LTPS* cDNAs into amino sequences generated 14, 7, and 2 amino sequences for LTPS-1, LTPS-3, and LTPS-2, respectively. The amino acid sequences from “Sorbonne” lily LsoTPS-1, LsoTPS-2, and LsoTPS-3 were used for characterization of the encoded proteins. All three sequences contained the highly conserved N-terminal arginine-rich RRX8W signature sequence motif and the C-terminal aspartate-rich DDXXD and NSE/DTE substrate-binding motifs^32^, with a predicted mass of ca. 66.3 kDa and a pI of ca. 5.8 (Fig. 4). The N-terminal 20 amino acids upstream of the conserved RRX8W motif were predicted to code for a transit peptide for import into plastids using the SignalP 4.1 peptide prediction tool. Although one highly conserved RX8W motif was found in the LsoTPS-1 and LsoTPS-2 amino sequences, a similar sequence was absent from the predicted LsoTPS-3 amino sequence (Fig. 4) because of the deletion produced by alternative splicing, as discussed above.

### Functional characterization of LTPS-1, LTPS-2, and LTPS-3

**Fig. 5:**
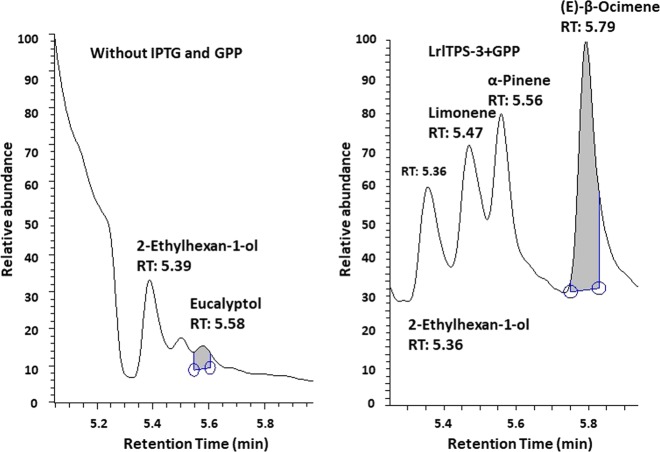
Representative results for GC-MS analysis of compounds generated by five *TPS* genes in *E. coli*. **a** Profile for a control group and the transformed *E. coli* cells cultivated in buffer without IPTG and GPP; **b** A representative profile following the transformation of the *LrlTPS-3* gene and the addition of IPTG and GPP. Since the five recombinant protein showed the same activity in converting GPP into three monoterpenes, only one representative profile is presented

To determine the functional activity of the three structurally distinct types of TPS proteins, five cDNAs were expressed separately in *Escherichia coli*. BL21, three from the scented lily *Lilium* cv. “Sorbonne” (*LsoTPS-1*, *LsoTPS-2*, and *LsoTPS-3*) and two from the faint-scented *Lilium* cv. “Red life” (*LrlTPS-1*, MH203231, and *LrlTPS-3*, MH203298). The recombinant plasmid with the full-length *LsoTPS-1*, *LsoTPS-2*, and *LsoTPS-3* cDNA sequences resulted in successful expression of soluble Lso*TPS*-1, Lso*TPS*-2, and Lso*TPS*-3 proteins of approximately 67, 68, and 62 kDa, respectively. The recombinant plasmid with the full-length *LrlTPS-1* and *LrlTPS-3* cDNAs resulted in successful expression of soluble proteins of approximately 67 and 62 kDa, respectively. Each of the purified proteins was tested for the ability to convert GPP into monoterpenes, and the products generated were analyzed by gas chromatograph–mass spectrometer (GC-MS). The results showed that all five proteins were able to convert GPP into (E)-*β*-ocimene, α-pinene, and limonene, which were identified by retention time at 5.79, 5.56, and 5.47 min, respectively (Fig. 5 shows the results for *LrlTPS-3* as an example). As shown in Fig. 5, even if the transformed *E. coli* cells were cultivated in buffer without isopropyl-β-d-thiogalactoside (IPTG) and substrate (GPP), a small amount of eucalyptol was produced, as identified by retention time at 5.58 min.

## Discussion

### Complexity and diverse nature of lily floral aroma compounds

Mixtures of volatile compounds are responsible for the varied fragrances of flowers and fruits. In this study, a diverse range of lily accessions was used for the analysis of floral volatiles and terpene synthase genes, including the largest lilies, new cultivars and hybrids, and scented and faint-scented types. A total of 46 volatile compounds were identified from 41 lily accessions, including 16 reported for the first time in lily (Supplementary Table 1). The observed differences between the volatiles reported here and the figures given by other authors^14,17,18^ can be explained either by the different flower scent collection methods employed or the different lily accessions investigated. Monoterpene hydrocarbons, monoterpene alcohols and aldehydes, phenylpropanoids, benzenoids, and fatty acid derivatives were the major chemical classes found (Supplementary Table 1).

Although it has been reported that monoterpenoids and benzenoids are the major compounds emitted from scented lily flowers^14,17^, volatiles such as eucalyptol, linalool, and benzenoids were also identified in some faint-scented lilies in this research. The level of gene expression might influence the production of important volatile organic compounds, but it is also possible that volatile compounds can be synthesized in some faint-scented lilies; however, obstacles in the secretary pathway may prohibit their emission in amounts that are sufficient for aroma generation. It is well known that floral scent compounds are produced in a number of diverse secretory compartments, including nectaries, glandular trichomes, eliaphores, osmophores^40,41^, and tepals, which are the source of lily floral scent^14,17^. So far, little is known in lilies or other flowers about the intracellular biosynthesis of volatile compounds in secretary cells and the trafficking of these compounds from their sites of synthesis to their sites of emission^42–44^. Those faint-scented lilies, such as “Black Eye” and “Pearl Melanie” (Table 2 and Supplementary Table 1), that emitted faint characteristic flower scent compounds would be suitable materials for further research on factors that limit the transportation of volatile organic components to sites where they can be emitted.

Classification of aroma types is challenging, and in many cases, it is difficult for the human nose to characterize the sensual features of a flower. The development of sensitive analytical methods, such as solid-phase microextraction (SPME), headspace adsorption, and GC-MS, has made it easier to collect and analyze volatile compounds, and these methods have been applied to research the aromas of liquor^45^, tobacco^46^, fruits such as strawberry^47^ and grape^48^, and flowers such as rose^49^ and tulip^13^. In most cases, flower aroma levels have been described simply as nonscented, light scented, and strong scented^17^. A more sophisticated method of classification by scent quality previously used for tulip cultivars^13^ was introduced in this study, and 41 lily cultivars were classified into six groups according to the composition of major scent components: Group 1, faint scented; Group 2, cool; Group 3, fruity; Group 4, musky; Group 5, fruity-honey; and Group 6, lily (Table 2). To our knowledge, this is the first time that lily cultivars have been classified by scent quality, and this precision may encourage successful efforts to breed fragrant lilies.

### Correlation between the nucleotide diversity of lily monoTPSs and the production of volatile compounds

Monoterpenes are major volatile constituents of many plants, such as rose^7^, grapevine^48^, and strawberry guava^50^. Nucleotide diversity is closely related to phenotypic polymorphism and has been applied in association studies^51^ and for haplotype mapping^52^ and linkage disequilibrium^53^. In this study, 32 candidate monoTPS cDNAs were obtained from 66 lily cultivars, and two InDels and 64 SNPs were identified. The nucleotide diversity for LTPS ranged from 0.001 (for LTPS-2) to 0.010 (for LTPS-1) (Table 4). Nucleotide diversity differs with species, cultivars, and genes. In an investigation of the nucleotide diversity of 11 frost-tolerance genes in five winter rye populations, *π* ranged from 0.004 to 0.0145^53^ and *π* was 0.00834 for *O*-methyltransferase genes in 42 maize inbred lines^54^.

As a mid-sized gene family, plant TPSs have been subclassified into seven subfamilies^55,56^. MonoTPSs belong to subfamilies TPS-b, TPS-g, and TPS-e/f^33,36^, and the size of the monoTPS gene family mirrors the complexity of monoterpene emissions from different plants. Phylogenetic analysis showed that the 32 candidate monoTPS cDNAs in lilies identified in the present study all belonged to the TPS-b subfamily (Supplementary Fig. 2). LTPS-1 presented the highest levels of nucleotide diversity in this study, and a total of 23 LTPS-1 were identified from 78.8% of lily cultivars, including the Asiatic hybrid “Little Kiss” (faint scented), Oriental hybrid “Sorbonne” (lily scent), OT hybrid “Pink Mist” (fruity-honey scent), Trumpet hybrid “Pink Perfection” (musky scent), and LA hybrid “Golden Stone” (fruity scent) (Tables 1–3). The two TPS sequences reported from the typical lily-scented “Siberia” and “Belladonna” by Zhang et al.^37^ and Johnson et al.^14^ are members of subfamily TPS-b and were similar/identical to TPS-1 in our study (Supplementary Fig. 2). Recently, Abbas et al.^57^ reported *LoTPS3* from “Siberia” belonging to the TPS-g family.

In lily, all identified nucleotide substitution sites were highly related to the amounts of myrcene emitted (Fig. 3), which varied from 0% to 14.67% in different lily accessions (Supplementary Table 1). InDel site 230 was found to be highly correlated with all emissions of major monoterpenoid components, especially (E)-*β*-ocimene (Fig. 3), which indicates the potential importance of this site for determining the production of monoterpenes. Unfortunately, the volatile compounds from lilies comprise such a complex mixture of aroma types (herbal, cool, fruity, etc.) present in different concentrations, with each volatile compound having its own threshold values (Supplementary Table 2), that the differences in (E)-*β*-ocimene and myrcene observed in this study cannot be linearly related to the differences in aroma. The alignment of genomic DNA sequences with those obtained from LTPS transcripts led to the discovery of variable splicing (Fig. 2), which indicates that the variation in emission might be regulated at the posttranscriptional level, as has been suggested previously for other plants^58^. It is well established that nearly all splice sites conform to consensus sequences with GT at the 5′ end of the intron and AG at the 3′ end of the intron^59^. These sequences were found at the alternative splice sites in this study and are indicated in Fig. 2c. The functional significance of this alternative splicing needs to be directly tested, however.

### MonoTPSs in lily encode multifunctional enzymes

Plant terpene synthases share a common evolutionary origin based on their similar reaction mechanisms and conserved structural and sequence characteristics^60^. All angiosperm monoterpene synthase genes contain six introns and seven exons^36,60^, and the TPS protein family is characterized by three domains: the N-terminal RRX8W motif and C-terminal DDXXD and NSE/DTE motifs^61^. Yueh-Te cloned three monoterpene synthase genes from *Litsea cubeba* that converted GPP into different products^55^. Shimada isolated four cDNA clones for monoterpene synthase genes from *Satsuma mandarin*, and each one encoded a different synthase^62^. In most lilies, however, only one monoTPS cDNA was cloned, although in the present study, two types of monoTPS cDNAs were cloned in eight lilies and three in “Sorbonne” (Table 3). The genomic *TPS* DNA cloned from “Sorbonne” lily contained seven exons and six introns, and the three conserved domains, RRX8W, DDXXD, and NSE/DTE, were also identified in the putative LTPS amino acid sequence (Figs. 2 and 4). Presumably, the cDNAs cloned in this study were from the same ancestral monoterpene synthase genes possessing the ability to catalyze the isomerization–cyclization reaction (RRX8W) and metal cofactor binding (DDXXD and NSE/DTE)^63^. The results of functional characterization experiments indicated that the enzymes encoded by monoTPS cDNAs from lily-scented “Sorbonne” and faint-scented “Red Life” could all catalyze the conversion of GPP into monoterpenes, which suggests that monoterpene production may be limited by substrate availability or transport in faint-scented flower types.

The striking feature of TPS enzymes is that a single TPS enzyme using a single substrate often gives rise to multiple products^32,34^. However, here, we showed that recombinant plasmids constructed from five cDNAs cloned from both faint- and strong-scented lilies were all multifunctional monoTPS that could convert GPP to (E)-*β*-ocimene, α-pinene, and limonene (Fig. 5). It has been reported that (E)-*β*-ocimene synthase in snapdragon produced three monoterpenes^64^. The protein encoded by *At3g25820*/*At3g25830* from *Arabidopsis* catalyzed the formation of ten volatile monoterpenes from GPP, as reported by Chen et al.^65^. It has been suggested that the formation of multiple products might be enhanced by the NSE/DTE motif^34^. However, the LsoTPS-3 and LrlTPS-3 forms both have catalytic activity in *E. coli*, but both lack the conserved RX8W motif, which suggests that this sequence is not essential for enzyme function or production of multiple products.

It is well known that monoterpene synthases have an N-terminal signal peptide that targets the initial translation product to the plastids^34,66^. Although several studies have highlighted interactions between plastidial MEP and cytosolic mevalonate pathways^10,67^, the occurrence of a predicted transit peptide indicates that monoterpenoids in *Lilium* might all be produced by the MEP pathway in plastids. Often, signal peptides are removed before *TPS* transcripts are expressed in *E. coli*^55^, but this did not occur in this study, which might explain why all *TPS*s in this study showed low catalytic activities when heterologously expressed (Fig. 5). The emission profiles from lily shown in Table 1 do not relate well to the emission measured after heterologous expression of the *TPS* genes in *E. coli*, and a comparison should be made of the catalytic activities with and without transit peptides. The emission results were obtained with 41 lilies of different aroma types, while *TPS*s from only two lilies were tested. In the latter case, the principal scent compounds in Table 2, apart from (E)-*β*-ocimene, did not correspond directly to the principal scent compounds produced during heterologous expression. More precise functional identification of alternatively spliced transcripts and their products will be the subject of a separate study.

In summary, we analyzed volatile emissions from 41 cultivars and performed a classification of flower aroma types of *Lilium*. In addition, we identified *Lilium* monoTPS polymorphisms and alternative splicing products and confirmed the catalytic activity of the encoded protein products. The findings in this study provide a major resource for the assessment of lily scent volatiles and will be helpful for the selection of breeding materials based on the volatile components they produce.

## Materials and methods

### Plant materials

Eighty-four *Lilium* accessions were planted in an open field at the Horticultural Station of Shanxi Agricultural University, Taigu, Shanxi, China on March 25, 2017, including 2 species (W), 27 Asiatic hybrids (A), 5 Trumpet hybrids (T), 6 Asiatic × Trumpet hybrids (AT), 1 Trumpet × Asiatic (TA), 15 Oriental × Trumpet hybrids (OT), 6 Trumpet × Oriental hybrids (TO), 5 Longiflorum × Asiatic hybrids (LA), 5 Longiflorum × Oriental hybrids (LO), 8 Oriental hybrids (O), 1 *L. formolongi* hybrid (F), 1 Longiflorum hybrid (L), 1 *Lilium longiflorum* × *L. pardalinum* hybrid (LP), and 1 Aurelian × *Lilium henryi* hybrid (AL) (Table 1). Petals were collected at the full flowering stage, frozen in liquid nitrogen, and stored at −80 °C for RNA extraction.

### Floral scent collection and analysis

Flowers of the 41 accessions listed in Table 1 were harvested at the full flowering stage from 8:00 to 10:00 a.m. on a sunny day from June to July 2018 for floral scent collection. Four or five accessions were selected from types for which there were more than five accessions. Triplicate samples were brought directly into the laboratory and used for sensory assessment and headspace volatile analysis. Volatile emission collections were conducted using the immersion SPME method^3^. Fresh flower samples (1.5 g) were placed in a 15 mL SPME vial, and 3-heptanone (Dr. Ehrensorfer Company with Limited Liability) was added as an internal standard. A preconditioned SPME fiber (50/30 μm PDMS/DVB/CAR, Supelco) was then exposed to the headspace of the capped vial at 50 °C for 15 min. The fiber was injected automatically and desorbed in the injection chamber of the GC in splitless mode for 5 min at 270 °C.

Quantification of volatiles was carried out using a GC-MS in the Sharing Platform of the Experimental Teaching Center of Shanxi Agricultural University. The GC (Trace ISQ, Thermo Scientific) was equipped with a capillary DB-5MS column (30 m × 0.25 mm inner diameter (i.d.) with 0.25-μm film thickness). Helium was used as the carrier gas, and the flow rate was 1 mL/min. The GC oven temperature was programmed at 40 °C for 2 min, increased from 40 to 270 °C at a rate of 5 °C/min, and then held for 5 min. The mass spectra (Trace ISQ, Thermo Scientific) were taken at electron ionization at 70 eV, and the mass range was 45–600 *m*/*z*. Both the ion source temperature and the transfer line temperature were 280 °C. Compounds were identified by matching to data from the NIST08 library in the Xcalibur software. Peak areas were normalized as a percentage and used to determine the amounts of the volatiles as described^68^: content of each component (μg/g) = [(peak area of each component × content of internal standard)/peak area of internal standard]/sample weight.

### Classification of scents

Odor description terms of fresh flowers were mainly derived by referring to Burdock^69^ and Oyama-Okubo^13^.

### RNA and DNA extraction and first-strand cDNA synthesis

The total RNA of a mixture of petals from the 66 lily cultivars in Table 1 was extracted following the TriGene reagent (GenStar) protocol. DNA from “Sorbonne” was isolated from young leaves using the CTAB method^70^. The quality and quantity of total RNA and genomic DNA were examined using a NanoDrop 2000C spectrophotometer (Thermo Scientific). First-strand cDNA was synthesized using the StarScript II First-Strand cDNA Synthesis Kit (GenStar) according to the manufacturer’s instructions and diluted to 100 ng/μL. The purified DNA samples were diluted to 20 ng/μL.

### Isolation of full-length *Lilium* monoTPS cDNA and genomic DNA

Cloning primers were designed based on the sequence of *LsTPS* (NCBI accession: MF401556) using Primer 3.0 (http://bioinfo.ut.ee/primer3-0.4.0/primer3/). Primer pairs (5′-ATGGCAGCTATGAGCTGT-3′/5′-TCATTCCAATGGGACATTATTG-3′) were synthesized by Sangon Biotech (Shanghai) Co., Ltd. PCRs were performed in 50 μL volumes: 2 μL of cDNA or DNA, 0.5 μL of each primer, 5 μL of 10 × TransTaq®-T Buffer, 4 μL of 2.5 mM dNTPs, 1 μL of TransTaq®-T DNA Polymerase, and 37 μL of ddH_2_O. The cycling conditions were as follows: 95 °C for 5 min, followed by 35 cycles of 95 °C for 30 s, 55 °C for 30 s, and 72 °C for 1 min. The final extension step was followed by incubation for 10 min at 72 °C. Gel extraction and purification of amplicons were carried out using a TIANgel Midi purification Kit (Tiangen Biotech). Each independent amplicon was ligated into the PMD-19T vector (TaKaRa) and then sequentially transformed into Trans5α chemically competent cells (TransGen Biotech). Sequencing was performed with three replicates by Sangon Biotech.

### Heterologous expression of monoterpene synthases

Restriction sites and protective bases were added to the 5′ end of the primer (5′-CGGACTAGTATGGCAGCTATGAGCTGT-3′/5′-CCGCTCGAGTCATTCCAATGGGACATTATTG-3′). The cloning process was the same as described above. Monoterpene synthase genes from lily-scented “Sorbonne” and faint-scented “Red life” were subcloned into the fusion protein expression vector pTYB12, and the resultant constructs were expressed in *E. coli* BL21(DE3) with intact plastidial signal peptides. The transformed *E. coli* cells were cultured overnight at 37 °C in Luria–Bertani medium and induced with 0.4 mM IPTG at 16 °C for 20 h. At the same time, the transformed *E. coli* cells were cultured overnight at 37 °C in Luria–Bertani medium and cultivated without 0.4 mM IPTG at 16 °C for 20 h as the control group (CK).

### Monoterpene synthase activity assay

The transformed *E. coli* cells were disrupted by a Bioruptor Plus sonication device. The product (mL) was added to monoTPS buffer (10 mM HEPES, pH 7.2; 100 mM KCl; 10 mM MgCl2; 10% (v/v) glycerol; 5 mM dithiothreitol) with 30 μM GPP (SinoStandards). At the same time, the product from CK was added to monoTPS buffer without GPP. The reaction mixture was incubated at 30 °C for 3 h. The products produced by the assay were collected using the immersion SPME method mentioned above and analyzed, with 3-heptanone as an internal standard, using a GC-MS equipped with a DB-5 capillary column (30 m length, 0.25 mm i.d., 0.25 μm film thickness). The oven temperature was held at 80 °C for 1 min, increased from 80 °C to 200 °C at a rate of 4 °C/min, and then held for 5 min. The injector temperature was 250 °C; the ion source temperature was 280 °C; the EI was 70 eV; the carrier gas was He at a flow rate of 1 mL/min; the mode was splitless; and the mass range was 45–425 m/z.

### Sequence analysis

Insertions, deletions, and SNP positions were assessed using DNAMAN 8. Hd of monoTPS was analyzed based on the SNPs in the amplified ORF sequences from 66 lily accessions. The levels of nucleotide diversity were estimated as *π*, the average number of nucleotide differences per site between two sequences^53^. Hd was estimated as the probability that two randomly chosen haplotypes from the 66 accessions were different^51,53^. Conserved domains in the protein were determined using NCBI Conserved Domains (http://www.ncbi.nlm.nih.gov/Structure/cdd/wrpsb.cgi). The phylogenetic trees were constructed using MEGA 6, and the bootstrap replication was 1000. SignalP 4.1 was used for transit peptide analysis (http://www.cbs.dtu.dk/services/SignalP/).

### Statistical analysis and data handling

Statistical analysis was carried out by IBM SPSS and least significant difference. Gray relational analysis was carried out by the DPS software^71^.

## Supplementary information

Supplementary Table 1: Forty-seven aroma compounds and their odor characteristics detected in flowers from 41 lily accessions.

Supplementary Fig. 1 Schematic representation of monoterpene biosynthesis via the plastidial methylerythritol phosphate (MEP) pathways.

Supplementary Fig. 2 Phylogeny of 32 putative lily TPS proteins and TPS proteins from other plants belonging to TPS-b, TPS-d, TPS-g and TPS-f analyzed using the UPGMA method of MEGA 6 software at the.

Supplementary Table 1 Forty-six aroma compounds and their odor characteristics detected in flowers from 41 lily accessions.

Supplementary Table 2 Descriptions and threshold values of major lily scent compounds.

Supplementary Table 3 Nucleotide polymorphisms in 32 terpene synthase cDNAs from Lilium.

Supplementary Sequence Data 32cDNA sequences from 66 lily accessions and one genomic DNA (gDNA) sequence from ‘Sorbonne’ lily.

## Data Availability

The raw data supporting the conclusions of this manuscript will be made available by the authors, without undue reservation, to any qualified researcher.
