# Supplementary figures and images for: Volatile composition and classification of Lilium flower aroma types and identification, polymorphisms, and alternative splicing of their monoterpene synthase genes
Source: Hortic Res. 2019 Oct 1;6:110. doi: 10.1038/s41438-019-0192-9 (PMC6804824; doi:10.1038/s41438-019-0192-9)

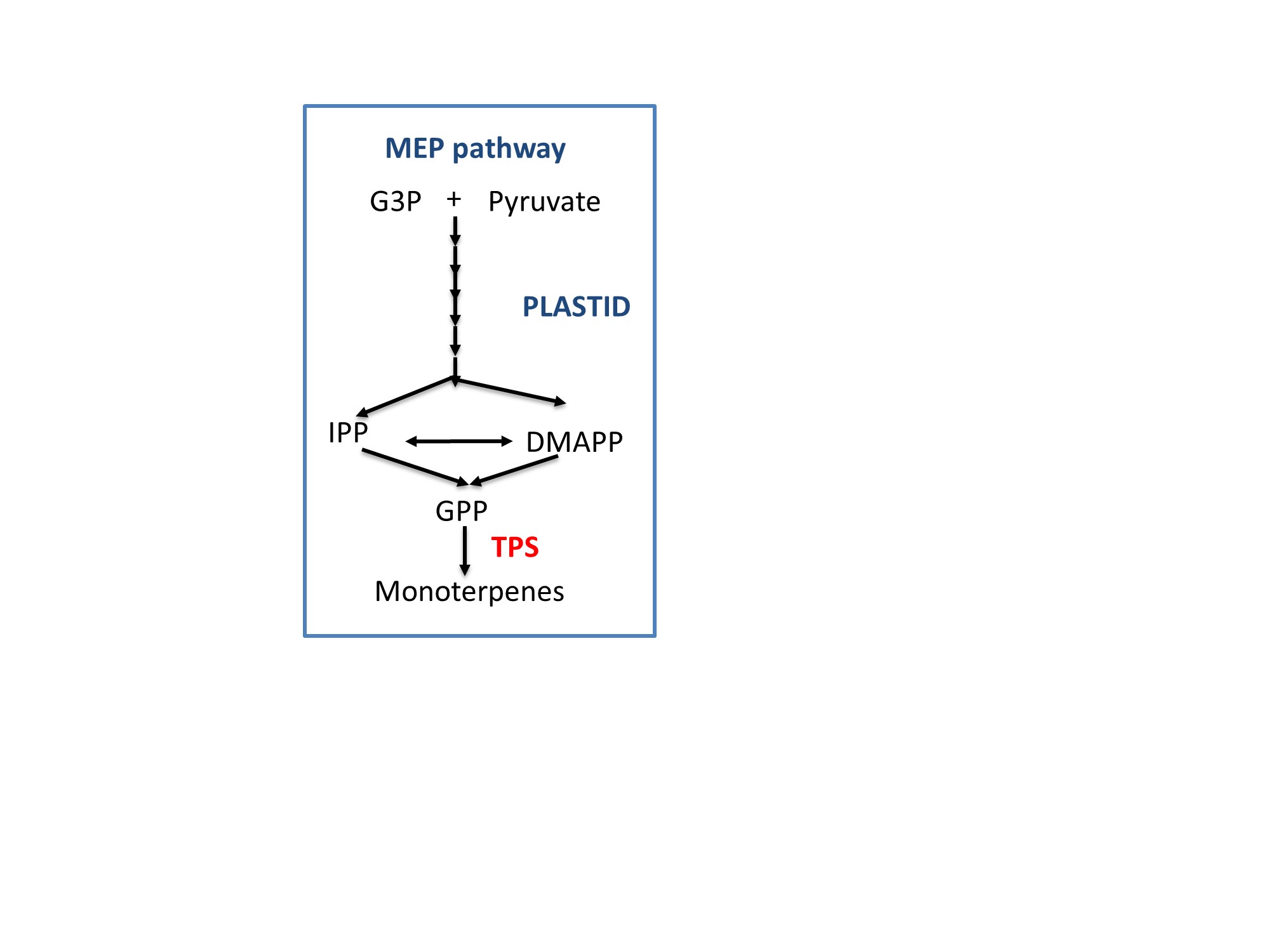

Supplement: Supplementary file 2 — Supplementary Fig. 1 Schematic representation of monoterpene biosynthesis via the plastidial methylerythritol phosphate (MEP) pathways. [file 41438_2019_192_MOESM2_ESM.jpg]

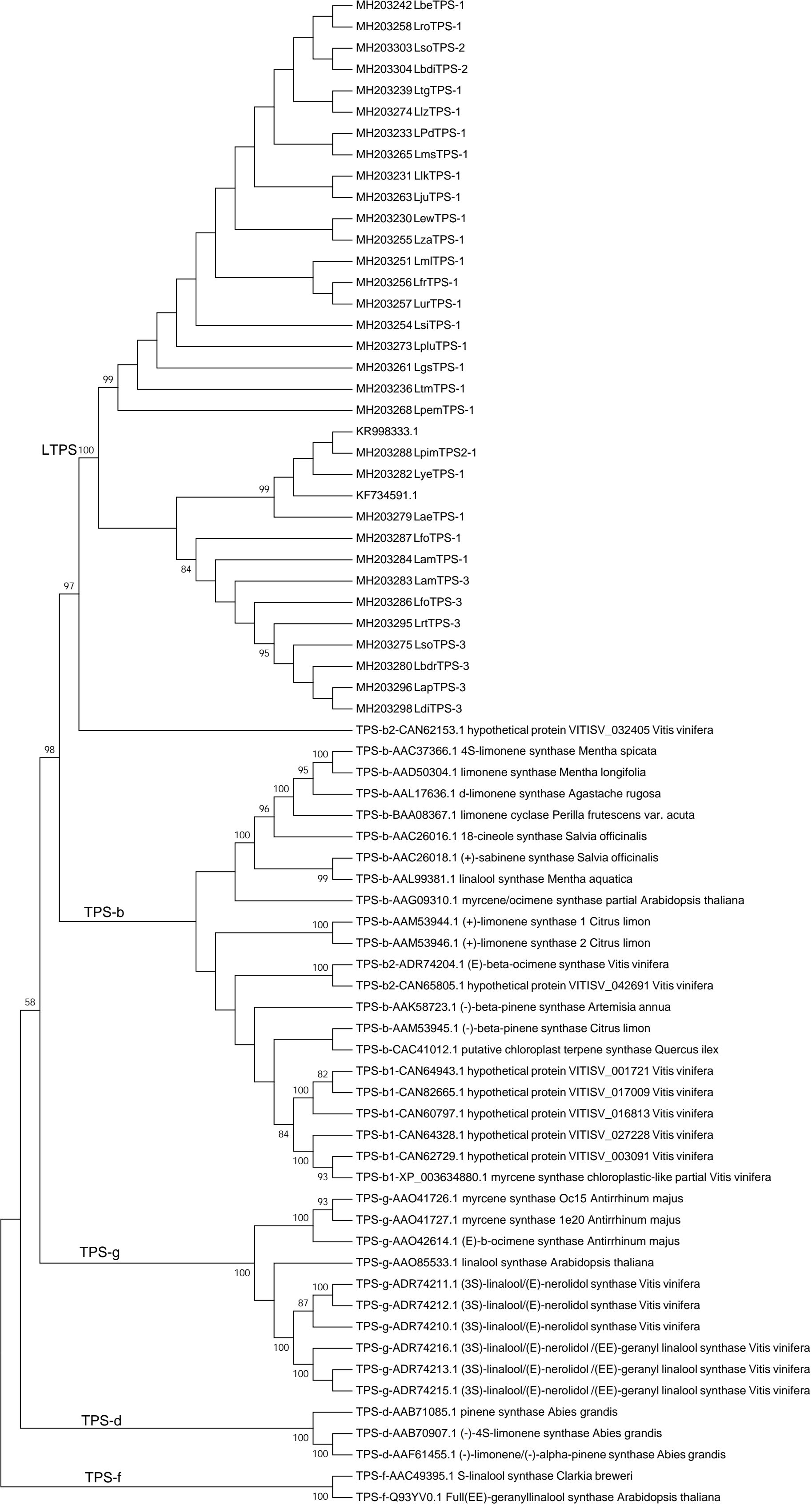

Supplement: Supplementary file 3 — Supplementary Fig. 2 Phylogeny of 32 putative lily TPS proteins and TPS proteins from other plants belonging to TPS-b, TPS-d, TPS-g and TPS-f analyzed using the UPGMA method of MEGA 6 software at the. [file 41438_2019_192_MOESM3_ESM.pdf]
