# Supplementary material for: Volatile composition and classification of Lilium flower aroma types and identification, polymorphisms, and alternative splicing of their monoterpene synthase genes
Source: Hortic Res. 2019 Oct 1;6:110. doi: 10.1038/s41438-019-0192-9 (PMC6804824; doi:10.1038/s41438-019-0192-9)
Supplement: Supplementary file 5 — Supplementary Table 2 Descriptions and threshold values of major lily scent compounds. [file 41438_2019_192_MOESM5_ESM.docx]

**Supplymentary Table 2**. Descriptions and threshold values of major lily scent compounds.

| **Type** | **Compounds** | **Descriptions** | **Threshold values** | **Odor classification** |
| --- | --- | --- | --- | --- |
| Monoterpenoid | (E)-β-Ocimene | Warm herbaceous odor^a^ | n/a | Herbal |
|  | Myrcene | Sweet-balsamic^b^ | 100ppb^d^ | Herbal |
|  | α-Pinene | Characteristic odor of pine^a^ | 2.5 to 65 ppb | Herbal |
|  | Eucalyptol | Characteristic camphoraceous odor and fresh, pungent, cooling taste^a^ | 1 to 64 ppb | Cool |
|  | Linalool | Typical pleasant floral odor^a^ | 4 to 10 ppb | Floral |
| Sesquiterpene | Caryopyhllene | Woody-spicy, dry, clove-like aroma^a^ | 64 to 90 ppb | Spicy |
| Benzenoids | Methyl benzoate | Fruity odor, similar to cananga^a^ | 110 ppb | Fruity^a^ |
|  | Ethyl benzoate | Somewhat fruity odor similar to ylang-ylang | 100 to 150 ppb | Fruity^c^ |
|  | Toluene | Paint thinner odor (Wikipedia) | n/a | n/a |
| Phenylpropanoid-related | Naphthalene | The main ingredient of traditional [mothballs](https://en.wikipedia.org/wiki/Mothball) (Wikipedia) | n/a | Cool |
|  | 3-Hexen-1-ol | Intense, grassy-green odor^e^ | 70 ppb | Herbal^c^ |
|  | Methyl 2-methylbutyrate | Sweet, fruity, apple-like odor^e^ | 0.92 to 4.4 ppb | Fruity^c^ |
|  | Methyl tiglate | n/a | n/a | n/a |
| Fatty acid derived | Methyl hexanoate | Ether-like odor reminiscent of pineapple^c^ | 10 to 87 ppb | Fruity ^c^ |
|  | Methyl octanoate | Winy, fruity and orange-like odor | 200 to 870 ppb | Fruity^c^ |
|  | 2-Ethenyl-1,1-dimethyl-3-methylenecyclohexane | A volatile constituent of the fruit of Garcinia atroviridis (Wikipedia) | n/a | Fruity |

^a^ Descriptions after Oyama-Okubo and Tsuji (2013). ^b^ From Pino et al. (2001). ^c^ From Feng et al. (2014). ^d^ From Liu et al. (2015). ^e^ From Burdock (2010).
