# Supplementary material for: Volatile composition and classification of Lilium flower aroma types and identification, polymorphisms, and alternative splicing of their monoterpene synthase genes
Source: Hortic Res. 2019 Oct 1;6:110. doi: 10.1038/s41438-019-0192-9 (PMC6804824; doi:10.1038/s41438-019-0192-9)
Supplement: Supplementary file 7 — Supplementary Sequence Data 32cDNA sequences from 66 lily accessions and one genomic DNA (gDNA) sequence from ‘Sorbonne’ lily. [file 41438_2019_192_MOESM7_ESM.docx]

>LlkTPS-1,MH203231

ATGGCAGCTATGAGCTGTTTCTCACTGGCCCGACTTCCCATCTCATCCACTTCTTCGAGTACTCGCAATTTCAGTTGTCTAACAAAACCTCATTCAATGCGGAGGTCATGCAAGATAGTCAATACTACGGAGAAATTGGAAGAACCTCCCCGTCGATCCGCCAATTACCACCCAACCATATGGGATCACTCGACCATCCAATCCATTGAGAATTTTAGTTCATTAAAGGGGAACACACTTGAAAGGCGAAGAGAATCACTTAAGAATGAAGTGAAGTTATTGCTTGATGCATCAGACGATCCAGTGGCTCAGCTCCAGCTCATTGACACTATTCAACGGCTTGGCATCGCCTACCATTTCGATAACGAGATTAAATCTATCCTTGATAGAATACGTGATTGTCATTTTGATCTAGAGGCTTTGGACTGTGTCCATAAGACGGCTCTTGCCTTTCGTCTACTCCGACAACATGGTTACGATGTGTCATCAGATGTTTTTCAGAAGTACAGAGACTCGCAAGGGTTCAAGGACTCCCTTACCGACGACGTGAAAGGACTGCTGAGCTTGTACGAAGCTTCTTTCCTTTCATTTCCAGGAGAACAACTGATGGAGGAAGCTAATAAGTTCAGCGTTAGGCACCTAGAGAGTTTGACTCAAAAGGTAGGGTTAGATATCGAGGAACAAGTGAGACACTCTCTACAAGTTCCATTGCATCGGAGGATGAGGAGACTCGAAGCTAGGGAGTACATAGATGTGTACCAGAGAGAGGAGGGGAAGAGCTCAGTCCTGCTAGAGTTTGCCAAGGTCGATTTCAACTTTGTGCAAATCATACATCAAATGGAATTGAAAGAACTCTCCAAATGGTGGATAAGCTTGAATTTGGGTAGTGTGCTCAGTTTTACCAGAGACAGATTAGTTGAAAATTATCTATGGGCTATCGGATTTGTCTATGAGCCCCATATGTCAAAATGTAGGATCGGCATCACTAAGGCGGTATGCATTTTATCCGTTATTGATGATGTCTATGATATATATGGGTCTTTTGAGGAAGTGGAGATCTTAACAAAGACGATCAAAAGTTGGGATCCTCATGAAATGAGAAACCTCCCCGAAAATATCAAGTTATGCTATAAGATACTTTACAATTTTATTGAGGAGATTACCACATGTACTCTATTGGATCATGGGTGCAATGTTATGCCCTTTCTAAAAGAAGAGTGGGAGAATTTATGTGGAGCATTTTTGGTAGAAGCAAAATGGTTCCATGAAGGCTATACTCCTTCTCTTAAAGAATACCTAAAGAATGCGTGGATTTCAATTGGTGGGCCGATGACCTTTGTGTTTGCTTATTGTCTCCTAGGTCACACATTGGGAGATAATTCCCTTAATTGCTTAAAACAAGGTTTCGATCCTATATACTGGTCTTCACTGATACTCCGTCTCAATGATGATTTGGGAACTTCTAAGGTCGAAATGGAAAGAGGAGACACACCCAAATCAATTCAATGCCATATGAGAGAAACAGATGAATCAGATGAGGTATCTAGAGAATACATAAAGAACTTAGTGGACCATTTTTGGAAGGAGTTGAATCAAGAATCTATAAGAACTCATCTTCCAAAGAATTTTATGAACTTAGTCACGAACATGGCCCTAGCATCTCACTGCATCTTCCAATTTGGAGATGGAATTGGAGATTCCACTGGCATAACAAAAAATCGTATCCTCTCACTATTTTTCAATAATGTCCCATTGGAATGA

>LewTPS-1,MH203230

ATGGCAGCTATGAGCTGTTTCTCACTGGCCCGACTTCCCATCTCATCCACTTCTTCGAGTACTCGCAATTTCAGTTGTCTAACAAAACCTCATTCAATGCGGAGGTCATGCAAGATAGTCAATACTACGGAGAAATTGGAAGAACCTCCCCGTCGATCCGCCAATTACCACCCAACCATATGGGATCACTCGACCATCCAATCCATTGAGAATTTTAGTTCATTAAAGGGGAACACACTTGAAAGGCGAAGAGAATCACTTAAGAATGAAGTGAAGTTATTGCTTGATGCATCAGACGATCCAGTGGCTCAGCTCCAGCTCATTGACACTATTCAACGGCTTGGCATCGCCTACCATTTCGATAACGAGATTAAATCTATCCTTGATAGAATACGTGATTGTCATTTTGATCTAGAGGCTTTGGACTGTGTCCATAAGACGGCTCTTGCCTTTCGTCTACTCCGACAACATGGTTACGATGTGTCATCAGATGTTTTTCAGAAGTACAGAGACTCGCAAGGGTTCAAGGACTCCCTTACCGACGACGTGAAAGGACTGCTGAGCTTGTACGAAGCTTCTTTCCTTTCATTCCCAGGAGAACAACTGATGGAGGAAGCTAATAAGTTCAGCGTTAGGCACCTAGAGAGTTTGACTCAAAAGGTAGGGTTAGATATCGAGGAACAAGTGAGACACTCTCTACAAGTTCCATTGCATCGGAGGATGAGGAGACTCGAAGCTAGGGAGTACATAGATGTGTACCAGAGAGAGGAGGGGAAGAGCTCAGTCCTGCTAGAGTTTGCCAAGGTCGATTTCAACTTTGTGCAAATCATACATCAAATGGAATTGAAAGAACTCTCCAAATGGTGGATAAGCTTGAATTTGGGTAGTGTGCTCAGTTTTACCAGAGACAGATTAGTTGAAAATTATCTATGGGCTATCGGATTTGTCTATGAGCCCCATATGTCAAAATGTAGGATCGGCATCACTAAGGCGGTATGCATTTTATCCGTTATTGATGATGTCTATGATATATATGGGTCTTTTGAGGAAGTGGAGATCTTAACAAAGACGATCAAAAGTTGGGATCCTCATGAAATGAGAAACCTCCCCGAAAATATCAAGTTATGCTATAAGATACTTTACAATTTTATTGAGGAGATTACCACATGTACTCTATTGGATCATGGGTGCAATGTTATGCCCTTTCTAAAAGAAGAGTGGGAGA

ATTTATGTGGAGCATTTTTGGTAGAAGCAAAATGGTTCCATGAAGGCTATACTCCTTCTCTTAAAGAATACCTAAAGAATGCGTGGATTTCAATTGGTGGGCCGATGACCTTTGTGTTTGCTTATTGTCTCCTAGGTCACACATTGGGAGATAATTCCCTTAATTGCTTAAAACAAGGTTTCGATCCTATATACTGGTCTTCACTGATACTCCGTCTCAATGATGATTTGGGAACTTCTAAGGTCGAAATGGAAAGAGGAGACACACCCAAATCAATTCAATGCCATATGAGAGAAACAGATGAATCAGATGAGGTATCTAGAGAATACATAAAGAACTTAGTGGACCATTTTTGGAAGGAGTTGAATCAAGAATCTATAAGAACTCATCTTCCAAAGAATTTTATGAACTTAGTCACGAACATGGCCCTAGCATCTCACTGCATCTTCCAATTTGGAGATGGAATTGGAGATTCCACTGGCATAACAAAAAATCGTATCCTCTCGCTATTTTTCAATAATGTCCCATTGGAATGA

>LtmTPS-1,MH203236

ATGGCAGCTATGAGCTGTTTCTCACTGGCCCGACTTCCCATCTCATCCACTTCTTCGAGTACTCGCAATTTCAGTTGTCTAACAAAACCTCATTCAATGCGGAGGTCATGCAAGATAGTCAATACTACGGAGAAATTGGAAGAACCTCCCCGTCGATCCGCCAATTACCACCCAACCATATGGGATCACTCGACCATCCAATCCATTGAGAATTTTAGTTCATTAAAGGGGAACACACTTGAAAGGCGAAGAGAATCACTTAAGAATGAAGTGAAGTTATTGCTTGATGCATCAGACGATCCAGTGGCTCAGCTCCAGCTCATTGACACTATTCAACGGCTTGGCATCGCCTACCATTTCGATAACGAGATTAAATCTATCCTTGATAGAATACGTGATTGTCATTTTGATCTAGAGGCTTTGGACTGTGTCCATAAGACGGCTCTTGCCTTTCGTCTACTCCGACAACATGGTTACGATGTGTCATCAGATGTTTTTCAGAAGTACAGAGACTCGCAAGGGTTCAAGGACTCCCTTACCGACGACGTGAAAGGACTGCTGAGCTTGTACGAAGCTTCTTTCCTTTCATTTCCAGGAGAACAACTGATGGAGGAGGCTAATAAGTTCAGCGTTAGGCACCTAGAGAGTTTGACTCAAAAGGTAGGGTTAGATATCGAGGAACAAGTGAGACACTCTCTACAAGTTCCATTGCATCGGAGGATGAGGAGACTCGAAGCTAGGGAGTACATAGATGTGTACCAGAGAGAGGAGGGGAAGAGCTCAGTCCTGCTAGAGTTTGCCAAGGTCGATTTCAACTTTGTGCAAATCATACATCAAATGGAATTGAAAGAACTCTCCAAATGGTGGATAAGCTTGAATTTGGGTAGTGTGCTCAGTTTTACCAGAGACAGATTAGTTGAAAATTATCTAAGGGCTATCGGATTTGTCTATGAGCCCCATATGTCAAAATGTAGGATCGGCATCACTAAGGCGGTATGCATTTTATCCGTTATTGATGATGTCTATGATATATATGGGTCTTTTGAGGAAGTGGAGATCTTAACAAAGACGATCAGAAGTTGGGATCCTCATGAAATGAGAAACCTCCCCGAAAATATCAAGTTATGCTATAAGATACTTTACAATTTTATTGAGGAGATTACCACATGTACTCTATTGGATCATGGGTGCAATGTTATGCCCTTTCTAAAAGAAGAGTGGGAGAATTTATGTGGAGCATTTTTGGTAGAAGCAAAATGGTTCCATGAAGGCTATACTCCTTCTCTTAAAGAATACCTAAAGAATGCGTGGATTTCAATTGGTGGGCCGATGACCTTTGTGTTTGCTTATTGTCTCCTAGGTCACACATTGGGAGATAATTCCCTTAATTGCTTAAAACAAGGTTTCGATCCTATATACTGGTCTTCACTGATACTCCGTCTCAATGATGATTTGGGAACTTCTAAGGTCGAAATGGAAAGAGGAGACACACCCAAATCAATTCAATGCCATATGAGAGAAACAGATGAATCAGATGAGGTATCTAGAGAATACATAAAGAACTTAGTGGACCATTTTTGGAAGGAGTTGAATCAAGAATCTATAAGAACTCATCTTCCAAAGAATTTTATGAACTTAGTCACGAACATGGCCCTAGCATCTCACTGCATCTTCCAATTTGGAGATGGAATTGGAGATTCCACTGGCATAACAAAAAATCGTATCCTCTCACTATTTTTCAATAATGTCCCATTGGAATGA

>LtgTPS-1,MH203239

ATGGCAGCTATGAGCTGTCTCTCACTGGCCCGACTTCCCATCTCATCCACTTCTTCGAGTACTCGCAATTTCAGTTGTCTAACAAAACCTCATTCAATGCGGAGGTCATGCAAGATAGTCAATACTACGGAGAAATTGGAAGAACCTCCCCGTCGATCCGCCAATTACCACCCAACCATATGGGATCACTCGACCATCCAATCCATTGAGAATTTTAGTTCATTAAAGGGGAACACACTTGAAAGGCGAAGAGAATCACTTAAGAATGAAGTGAAGTTATTGCTTGATGCATCAGACGATCCAGTGGCTCAGCTCCAGCTCATTGACACTATTCAACGGCTTGGCATCGCCTACCATTTCGATAACGAGATTAAATCTATCCTTGATAGAATACGTGATTGTCATTTTGATCTAGAGGCTTTGGACTGTGTCCATAAGACGGCTCTTGCCTTTCGTCTACTCCGACAACATGGTTACGATGTGTCATCAGATGTTTTTCAGAAGTACAGAGACTCGCAAGGGTTCAAGGACTCCCTTACCGACGACGTGAAAGGACTGCTGAGCTTGTACGAAGCTTCTTTCCTTTCATTTCCAGGAGAACAACTGATGGAGGAAGCTAATAAGTTCAGCGTTAGGCACCTAGAGAGTTTGACTCAAAAGGTAGGGTTAGATATCGAGGAACAAGTGAGACACTCTCTACAAGTTCCATTGCATCGGAGGATGAGGAGACTCGAAGCTAGGGAGTACATAGATGTGTACCAGAGAGAGGAGGGGAAGAGCTCAGTCCTGCTAGAGTTTGCCAAGGTCGATTTCAACTTTGTGCAAATCATACATCAAATGGAATTGAAAGAACTCTCCAAATGGTGGATAAGCTTGAATTTGGGTAGTGTGCTCAGTTTTACCAGAGACAGATTAGTTGAAAATTATCTATGGGCTATCGGATTTGTCTATGAGCCCCATATGTCAAAATGTAGGATCGGCATCACTAAGGCGGTATGCATTTTATCCGTTATTGATGATGTCTATGATATATATGGGTCTTTTGAGGAAGTGGAGATCTTAACAAAGACGATCAAAAGTTGGGATCCTCATGAAATGAGAAACCTCCCCGAAAATATCAAGTTATGCTATAAGATACTTTACAATTTTATTGAGGAGATTACCACATGTACTCTATTGGATCATGGGTGCAATGTTATGCCCTTTCTAAAAGAAGAGTGGGAGAATTTATGTGGAGCATTTTTGGTAGAAGCAAAATGGTTCCATGAAGGCTATACTCCTTCTCTTAAAGAATACCTAAAGAATGCGTGGATTTCAATTGGTGGGCCGATGACCTTTGTGTTTGCTTATTGTCTCCTAGGTCACACATTGGGAGATAATTCCCTTAATTGCTTAAAACAAGGTTTCGATCCTATATACTGGTCTTCACTGATACTCCGTCTCAATGATGATTTGGGAACTTCTAAGGTCGAAATGGAAAGAGGAGACACACCCAAATCAATTCAATGCCATATGAGAGAAACAGATGAATCAGATGAGGTATCTAGAGAATACATAAAGAACTTAGTGGACCATTTTTGGAAGGAGTTGAATCAAGAATCTATAAGAACTCATCTTCCAAAGAATTTTATGAACTTAGTCACGAACATGGCCCTAGCATCTCACTGCATCTTCCAATTTGGAGATGGAATTGGAGATTCCACTGGCATAACAAAAAATCGTATCCTCTCACTATTTTTCAATAATGTCCCATTGGAATGA

>LpluTPS-1,MH203273

ATGGCAGCTATGAGCTGTTTCTCACTGGCCCGACTTCCCATCTCATCCACTTCTTCGAGTACTCGCAATTTCAGTTGTCTAACAAAACCTCATTCAATGCGGAGGTCATGCAAGATAGTCAATACTACGGAGAAATTGGAAGAACCTCCCCGTCGATCCGCCAATTACCACCCAACCATATGGGATCACTCGACCATCCAATCCATTGAGAATTTTAGTTCATTAAAGGGGAACACACTTGAAAGGCGGAGAGAATCACTTAAGAATGAAGTGAAGTTATTGCTTGATGCATCAGACGATCCAGTGGCTCAGCTCCAGCTCATTGACACTATTCAACGGCTTGGCATCGCCTACCATTTCGATAACGAGATTAAATCTATCCTTGATAGAATACGTGATTGTCATTTTGATCTAGAGGCTTTGGACTGTGTCCATAAGACGGCTCTTGCCTTTCGTCTACTCCGACAACATGGTTACGATGTGTCATCAGATGTTTTTCAGAAGTACAGAGACTCGCAAGGGTTCAAGGACTCCCTTACCGACGACGTGAAAGGACTGCTGAGCTTGTACGAAGCTTCTTTCCTTTCATTTCCAGGAGAACAACTGATGGAGGAAGCTAATAAGTTCAGCGTTAGGCACCTAGAGAGTTTGACTCAAAAGGTAGGGTTAGATATCGAGGAACAAGTGAGACACTCTCTACAAGTTCCATTGCATCGGAGGATGAGGAGACTCGAAGCTAGGGAGTACATAGATGTGTACCAGAGAGAGGAGGGGAAGAGCTCAGTCCTGCTAGAGTTTGCCAAGGTCGATTTCAACTTTGTGCAAATCATACATCAAATGGAATTGAAAGAACTCTCCAAATGGTGGATAAGCTTGAATTTGGGTAGTGTGCTCAGTTTTACCAGAGACAGATTAGTTGAAAATTATCTATGGGCTATCGGATTTGTCTATGAGCCCCATATGTCAAAATGTAGGATCGGCATCACTAAGGCGGTATGCATTTTATCCGTTATTGATGATGTCTATGATATATATGGGTCTTTTGAGGAAGTGGAGATCTTAACAAAGACGATCAAAAGTTGGGATCCTCATGAAATGAGAAACCTCCCCGAAAATATCAAGTTATGCTATAAGATACTTTACAATTTTATTGAGGAGATTACCACATGTACTCTATTGGATCGTGGGTGCAATGTTATGCCCTTTCTAAAAGAAGAGTGGGAGAATTTATGTGGAGCATTTTTGGTAGAAGCAAAATGGTTCCATGAAGGCTATACTCCTTCTCTTAAAGAATACCTAAAGAATGCGTGGATTTCAATTGGTGGGCCGATGACCTTTGTGTTTGCTTATTGTCTCCTAGGTCACACATTGGGAGATAATTCCCTTAATTGCTTAAAACAAGGTTTCGATCCTATATACTGGTCTTCACTGATACTCCGTCTCAATGATGATTTGGGAACTTCTAAGGTCGAAATGGAAAGAGGAGACACACCCAAATCAATTCAATGCCATATGAGAGAAACAGATGAATCAGATGAGGTATCTAGAGAATACATAAAGAACTTAGTGGACCATTTTTGGAAGGAGTTGAATCAAGAATCTATAAGAACTCATCTTCCAAAGAATTTTATGAACTTAGTCACGAACATGGCCCTAGCATCTCACTGCATCTTCCAATTTGGAGATGGAATTGGAGATTCCACTGGCATAACAAAAAATCGTATCCTCTCACTATTTTCCAATAATGTCCCATTGGAATGA

>LlzTPS-1,MH203274

ATGGCAGCTATGAGCTGTTTCTCACTGGCCCGACTTCCCATCTCATCCACTTCTTCGAGTACTCGCAATTTCAGTTGTCTAACAAAACCTCATTCAATGCGGAGGTCATGCAAGATAGTCAATACTACGGAGAAATTGGAAGAACCTCCCCGTCGATCCGCCAATTACCACCCAACCATATGGGATCACTCGACCATCCAATCCATTGAGAATTTTAGTTCATTAAAGGGGAACACACTTGAAAGGCGAAGAGAATCACTTAAGAATGAAGTGAAGTTATTGCTTGATGCATCAGACGATCCAGTGGCTCAGCTCCAGCTCATTGGCACTATTCAACGGCTTGGCATCGCCTACCATTTCGATAACGAGATTAAATCTATCCTTGATAGAATACGTGATTGTCATTTTGATCTAGAGGCTTTGGACTGTGTCCATAAGACGGCTCTTGCCTTTCGTCTACTCCGACAACATGGTTACGATGTGTCATCAGATGTTTTTCAGAAGTACAGAGACTCGCAAGGGTTCAAGGACTCCCTTACCGACGACGTGAAAGGACTGCTGAGCTTGTACGAAGCTTCTTTCCTTTCATTTCCAGGAGAACAACTGATGGAGGAAGCTAATAAGTTCAGCGTTAGGCACCTAGAGAGTTTGACTCAAAAGGTAGGGTTAGATATCGAGGAACAAGTGAGACACTCTCTACAAGTTCCATTGCATCGGAGGATGAGGAGACTCGAAGCTAGGGAGTACATAGATGTGTACCAGAGAGAGGAGGGGAAGAGCTCAGTCCTGCTAGAGTTTGCCAAGGTCGATTTCAACTTTGTGCAAATCATACATCAAATGGAATTGAAAGAACTCTCCAAATGGTGGATAAGCTTGAATTTGGGTAGTGTGCTCAGTTTTACCAGAGACAGATTAGTTGAAAATTATCTATGGGCTATCGGATTTGTCTATGAGCCCCATATGTCAAAATGTAGGATCGGCATCACTAAGGCGGTATGCATTTTATCCGTTATTGATGATGTCTATGATATATATGGGTCTTTTGAGGAAGTGGAGATCTTAACAAAGACGATCAAAAGTTGGGATCCTCATGAAATGAGAAACCTCCCCGAAAATATCAAGTTATGCTATAAGATACTTTACAATTTTATTGAGGAGATTACCACATGTACTCTATTGGATCATGGGTGCAATGTTATGCCCTTTCTAAAAGAAGAGTGGGAGAATATATGTGGAGCATTTTTGGTAGAAGCAAAATGGTTCCATGGAGGCTATACTCCTTCTCTTAAAGAATACCTAAAGAATGCGTGGATTTCAATTGGTGGGCCGATGACCTTTGTGTTTGCTTATTGTCTCCTAGGTCACACATTGGGAGATAATTCCCTTAATTGCTTAAAACAAGGTTTCGATCCTATATACTGGTCTTCACTGATACTCCGTCTCAATGATGATTTGGGAACTTCTAAGGTCGAAATGGAAAGAGGAGACACACCCAAATCAATTCAATGCCATATGAGAGAAACAGATGAATCAGATGAGGTATCTAGAGAATACATAAAGAACTTAGTGGACCATTTTTGGAAGGAGTTGAATCAAGAATCTATAAGAACTCATCTTCCAAAGAATTTTATGAACTTAGTCACGAACATGGCCCTAGCATCTCACTGCATCTTCCAATTTGGAGATGGAATTGGAGATTCCACTGGCATAACAAAAAATCGTATCCTCTCACTATTTTTCAATAATGTCCCATTGGAATGA

>LpemTPS-1,MH203268

ATGGCAGCTATGAGCTGTTTCTCACTGGCCCGACTTCCCATCTCATCCACTTCTTCGAGTACTCGCAATTTCAGTTGTCTAACAAAACCTCATTCAATGCGGAGGTCATGCAAGATAGTCAATACTACGGAGAAATTGGAAGAACCTCCCCGTCGATCCGCCAATTACCACCCAACCATATGGGATCACTCGACCATCCAATCCATTGAGAATTTTAGTTCATTAAAGGGGAACACACTTGGAAGGCGAAGAGAATCACTTAAGAATGAAGTGAAGTTATTGCTTGATGCATCAGACGATCCAGTGGCTCAGCTCCAGCTCATTGACACTATTCAACGGCTTGGCATCGCCTACCATTTCGATAACGAGATTAAATCTATCCTTGATAGAATACGTGATTGTCATTTTGATCTAGAGGCTTTGGACTGTGTCCATAAGACGGCTCTTGCCTTTCGTCTACTCCGACAACATGGTTACGATGTGTCATCAGATGTTTTTCAGAAGTACAGAGACTCGCAAGGGTTCAAGGACTCCCTTACCGACGACGTGAAAGGACTGCTGAGCTTGTACGAAGCTTCTTTCCTTTCATTTCCAGGAGAACAACTGATGGAGGAAGCTAATAAGTTCAGCGTTAGGCACCTAGAGAGTTTGACTCAAAAGGTAGGGTTAGATATCGAGGAACAAGTGAGACACTCTCTACAAGTTCCATTGCATCGGAGGATGAGGAGACTCGAAGCTAGGGAGTACATAGATGTGTACCAGAGAGAGGAGGGGAAGAGCTCAGTCCTGCTAGAGTTTGCCAAGGTCGATTTCAACTTTGTGCAAATCATACATCAAATGGAATTGAAAGAACTCTCCAAATGGTGGATAAGCTTGAATTTGGGTAGTGTGCTCAGTTTTACCAGAGACAGATTAGTTGAAAATTATCTATGGGCTATCGGATTTGTCTATGAGCCCCATATGTCAAAATGTAGGATCGGCATCACTAAGGCGGTATGCATTTTATCCGTTATTGATGATGTCTATGATATATATGGGTCCTTTGAGGAAGTGGAGATCTTAACAAAGACGATCAAAAGTTGGGATCCTCATGAAATGAGAAACCTCCCCGAAAATATCAAGTTATGCTATAAGATACTTTACAATTTTATTGAGGAGATTACCACATGTACTCTATTGGATCATGGGTGCAATGTTATGCCCTTTCTAAAAGAAGAGTGGGAGAATTTATGTGGAGCATTTTTGGTAGAAGCAAAATGGTTCCATGAAGGCTATACTCCTTCTCTTAAAGAATACCTAAAGAATGCGTGGATTTCAATTGGTGGGCCGATGACCTTTGTGTTTGCTTATTGTCTCCTAGGTCACACATTGGGAGATAATTCCCTTAATTGCTTAAAACAAGGTTTCGATCCTATATACTGGTCTTCACTGATACTCCGTCTCGATGATGATTTGGGAACTTCTAAGGTCGAAATGGAAAGAGGAGACACACCCAAATCAATTCAATGCCATATGAGAGAAACAGATGAATCAGATGAGGTATCTAGAGAATACATAAAGAACTTAGTGGACCATTTTTGGAAGGAGTTGAATCAAGAATCTATAAGAACTCATCTTCCAAAGAATTTTATGAACTTAGTCACGAACATGGCCCTAGCATCTCACTGCATCTTCCAATTTGGAGATGGAATTGGAGATTCCACTGGCATAACAAAAAATCGTATCCTCTCACTATTTTTCAATAATGTCCCATTGGAATGA

>LmlTPS-1,MH203251

ATGGCAGCTATGAGCTGTTTCTCACTGGCCCGACTTCCCATCTCATCCACTTCTTCGAGTACTCGCAATTTCAGTTGTCTAACAAAACCTCATTCAATGCGGAGGTCATGCAAGATAGTCAATACTACGGAGAAATTGGAAGAACCTCCCCGTCGATCCGCCAATTACCACCCAACCATATGGGATCACTCGACCATCCAATCCATTGAGAATTTTAGTTCATTAAAGGGGAACACACTTGAAAGGCGAAGAGAATCACTTAAGAATGAAGTGAAGTTATTGCTTGATGCATCAGACGATCCAGTGGCTCAGCTCCAGCTCATTGACACTATTCAACGGCTTGGCATCGCCTACCATTTCGATAACGAGATTAAATCTATCCTTGATAGAATACGTGATTGTCATTTTGATCTAGAGGCTTTGGACTGTGTCCATAAGACGGCTCTTGCCTTTCGTCTACTCCGACAACATGGTTACGATGTGTCATCAGATGTTTTTCAGAAGTACAGAGACTCGCAAGGGTTCAAGGACTCCCTTACCGACGACGTGAAAGGACTGCTGAGCTTGTACGAAGCTTCTTTCCTTTCATTTCCAGGAGAACAACTGATGGAGGAAGCTAATAAGTTCAGCGTTAGGCACCTAGAGAGTTTGACTCAAAAGGTAGGGTTAGATATCGAGGAACAAGTGAGACACTCTCTACAAGTTCCATTGCATCGGAGGATGAGGAGACTCGAAGCTAGGGAGTACATAGATGTGTACCAGAGAGAGGAGGGGAAGAGCTCAGTCCTGCTAGAGTTTGCCAAGGTCGATTTCAACTTTGTGCAAATCATACATCAAATGGAATTGAAAGAACTCTCCAAATGGTTGATAAGCTTGAATTTGGGTAGTGTGCTCAGTTTTACCAGGGACAGATTAGTTGAAAATTATCTATGGGCTATCGGATTTGTCTATGAGCCCCATATGTCAAAATGTAGGATCGGCATCACTAAGGCGGTATGCATTTTATCCGTTATTGATGATGTCTATGATATATATGGGTCTTTTGAGGAAGTGGAGATCTTAACAAAGACGATCAAAAGTTGGGATCCTCATGAAATGAGAAACCTCCCCGAAAATATCAAGTTATGCTATAAGATACTTTACAATTTTATTGAGGAGATTACCACATGTACTCTATTGGATCATGGGTGCAATGTTATGCCCTTTCTAAAAGAAGAGTGGGAGAATTTATGTGGAGCATTTTTGGTAGAAGCAAAATGGTTCCATGAAGGCTATACTCCTTCTCTTAAAGAATACCTAAAGAATGCGTGGATTTCAATTGGTGGGCCGATGACCTTTGTGTTTGCTTATTGTCTCCTAGGTCACACATTGGGAGATAATTCCCTTAATTGCTTAAAACAAGGTTTCGATCCTATATACTGGTCTTCACTGATACTCCGTCTCAATGATGATTTGGGAACTTCTAAGGTCGAAATGGAAAGAGGAGACACACCCAAATCAATTCAATGCCATATGAGAGAAACAGATGAATCAGATGAGGTATCTAGAGAATACATAAAGAACTTAGTGGACCATTTTTGGAAGGAGTTGAATCAAGAATCTATAAGAACTCATCTTCCAAAGAATTTTATGAACTTAGTCACGAACATGGCCCTAGCATCTCACTGCATCTTCCAATTTGGAGATGGAATTGGAGATTCCACTAGCATAACAAAAAATCGTATCCTCTCACTATTTTTCAATAATGTCCCATTGGAATGA

>LyeTPS-1,MH203282

ATGGCAGCTATGAGCTGTTTCTCACTGGCCCGACTTCCCATCTCATCCACTTCTTCGAGTACTCGCAATTTCAGTTGTCTAACAAAACCTCATTCAATTCAGAGGTCATGCAAGATAGTCAATACTACGGAGAAATTGGAAGAACCTCCCCGTCGATCCGCCAATTACCACCCAACCATATGGGATCACTCGACCATCCAATCCATTGAGAGTTTGAGTTCATTAAAGGGGAACACACTTGAAAGGCGAAGAGAATTACTTAAGAATGAAGTGAAGTTATTGCTTGATGCATCAGACGATCCAGTGGCTCAGCTCCATCTCATTGACACTATTCAACGGCTTGGCATCGCCTACCATTTTGATAACGAGATTAAATCTATCCTTGATAGAATACATGATTGTCATGTTGATCTAGAGGCTTTGGACTGTGTCCATAAGACGGCTCTTGCCTTTCGTCTACTCCGACAACATGGTTACGATGTGTCATCAGATGTTTTTCAGAAGTACAGAGACTCGCAAGGGTTCAAGGACTCCCTTACCGACGACGTGAAAGGACTGCTGAGCTTGTACGAAGCTTCTTTCCTTTCATTCCCAGGAGAACAACTGATGGAGGAAGCTAATAAGTTCAGCGTTAGGCACCTAGAGAGTTTGACTCAAAAGGTAGGGTTAGATATCGAGGAACAAGTGAGACACTCTCTACAGGTTCCATTGCACCGTAGGATGAGGAGACTCGAAGCTAGGGAGTACATAGATGTGTACCAGAGGGAGGAGGGGAAGAGCTCAGTCCTGCTAGAGTTTGCCAAGGTCGATTTCAACTTTGTGCAAATCATACATCAAATGGAATTGAAAGAACTCTCCAAATGGTGGATAAGCTTGAATTTGGGCAGTGTGCTCAGTTTCACAAGAGACAGATTAGTTGAAAATTATCTATGGGCTATCGGATTAGTCTATGAGCCCCATATGTCAAAATGTAGGATCGGCATCACTAAGGCAGTATGCATTTTATCCGTTATTGATGATGTCTATGATATTTATGGGTCTTTTGAGGAAGCGGAGATCTTAACAAAGACGATCAAAAGTTGGGATCCTCATGAAATGAGAAACCTCCCCGAAAATATCAAGTTATGCTATAAGATAATTTACAATTTTATTGAGGAGATTACCACATCTATTCTATTGGATTATGGGTGCAATGTTATGCCCTTTCTAAAAAAAGAGTGGGAGAATTTATGTGGAGCATATTTGGTAGAAGCAAAATGGTTCCATGAAGGCTATACTCCTTCTCTTAAAGAATACCTAAAGAATGCGTGGATTTCAATTGGTGGGCCGATGACCTTTGTTTTTGCTTATTGTCTCCTAGGACACACATTGGGAGATAATTCCCTTAATTGCTTAAAACAAGGTTTCGATCCTATATATTGGTCTTCACTAATACTCCGTCTCAATGATGATTTGGGAACTTCTAAGGCCGAAATGGAAAGAGGAGACACACCCAAATCAATTCAATGCCATATGAGAGAAACATGTGAATTAGAAGAGGTATCTAGAGAATACATAAAGAACTTAGTGGACCATTTTTGGAAGGAGTTGAATCAAAAATCTATAAGAACTCATCTTCCGAAGAATTTTATGAACTTAGTCACAAACATGGCCCTAGCATCTCACTGCATTTTCCAATTTGGAGATGGAATTGGAGATTCCACTGGCATAACAAAAAATCGTATCCTCTCACTATTTGTCAATAATGTCCCATTGGAATGA

>LpimTPS2-1,MH203288

ATGGCAGCTATGAGCTGTCTCTCACTGGCCCGACTTCCCATCTCATCCACTTCTTCGAGTACTCGCAATTTCAGTTGTCTAACAAAACCTCATTCAATTCAGAGGTCATGCAAGATAGTCAATACTACGGAGAAATTGGAAGAACCTCCCCGTCGATCCGCCAATTACCACCCAACCATATGGGATCACTCGACCATCCAATCCATTGAGAGTTTGAGTTCATTAAAGGGGAACACACTTGAAAGGCGAAGAGAATTACTTAAGAATGAAGTGAAGTTATTGCTTGATGCATCAGACGATCCAGTGGCTCAGCTCCATCTCATTGACACTATTCAACGGCTTGGCATCGCCTACCATTTTGATAACGAGATTAAATCTATCCTGGATAGAATACATGATTGTCATGTTGATCTAGAGGCTTTGGACTGTGTCCATAAGACGGCTCTTGCCTTTCGTCTACTCCGACAACATGGTTACGATGTGTCATCAGATGTTTTTCAGAAGTACAGAGACTCGCAAGGGTTCAAGGACTCCCTTACCGACGACGTGAAAGGACTGCTGAGCTTGTACGAAGCTTCTTTCCTTTCATTCCCAGGAGAACAACTGATGGAGGAAGCTAATAAGTTCAGCGTTAGGCACCTAGAGAGTTTGACTCAAAAGGTAGGGTTAGATATCGAGGAACAAGTGAGACACTCTCTACAGGTTCCATTGCACCGTAGGATGAGGAGACTCGAAGCTAGGGAGTACATAGATGTGTACCAGAGGGAGGAGGGGAAGAGCTCAGTCCTGCTAGAGTTTGCCAAGGTCGATTTCAACTTTGTGCAAATCATACATCAAATGGAATTGAAAGAACTCTCCAAATGGTGGATAAGCTTGAATTTGGGCAGTGTGCTCAGTTTCACAAGAGACAGATTAGTTGAAAATTATCTATGGGCTATCGGATTAGTCTATGAGCCCCATATGTCAAAATGTAGGATCGGCATCACTAAGGCAGTATGCATTTTATCCGTTATTGATGATGTCTATGATATTTATGGGTCTTTTGAGGAAGCGGAGATCTTAACAAAGACGATCAAAAGTTGGGATCCTCATGAAATGAGAAACCTCCCCGAAAATATCAAGTTATGCTATAAGATAATTTACAATTTTATTGAGGAGATTACCACATCTATTCTATTGGATTATGGGTGCAATGTTATGCCCTTTCTAAAAAAAGAGTGGGAGAATTTATGTGGAGCATATTTGGTAGAAGCAAAATGGTTCCATGAAGGCTATACTCCTTCTCTTAAAGAATACCTAAAGAATGCGTGGATTTCAATTGGTGGGCCGATGACCTTTGTTTTTGCTTATTGTCTCCTAGGACACACATTGGGAGATAATTCCCTTAATTGCTTAAAACAAGGTTTCGATCCTATATATTGGTCTTCACTAATACTCCGTCTCAATGATGATTTGGGAACTTCTAAGGCCGAAATGGAAAGAGGAGACACACCCAAATCAATTCAATGCCATATGAGAGAAACATGTGAATTAGAAGAGGTATCTAGAGAATACATAAAGAACTTAGTGGACCATTTTTGGAAGGAGTTGAATCAAAAATCTATAAGAACTCATCTTCCGAAGAATTTTATGAACTTAGTCACAAACATGGCCCTAGCATCTCACTGCATTTTCCAATTTGGAGATGGAATTGGAGATTCCACTGGCATAACAAAAAATCGTATCCTCTCACTATTTGTCAATAATGTCCCATTGGAATGA

>LaeTPS-1,MH203279

ATGGCAGCTATGAGCTGTTTCTCACTGGCCCGACTTCCCATCTCATCCACTTCTTCGAGTACTCGCAATTTCAGTTGTCTAACAAAACCTCATTCAATTCAGAGGTCATGCAAGATAGTCAATACTACGGAGAAATTGGAAGAACCTCCCCGTCGATCCGCCAATTACCACCCAACCATATGGGATCACTCGACCATCCAATCCATTGAGAGTTTGAGTTCATTAAAGGGGAACACACTTGAAAGGCGAAGAGAATTACTTAAGAATGAAGTGAAGTTATTGCTTGATGCATCAGACGATCCAGTGGCTCAGCTCCAGCTCATTGACACTATTCAACGGCTTGGCATCGCCTACCATTTTGATAACGAGATTAAATCTATCCTTGATAGAATACATGATTGTCATGTTGATCTAGAGGCTTTGGACTGTGTCCATAAGACGGCTCTTGCCTTTCGTCTACTCCGACAACATGGTTACGATGTGTCATCAGATGTTTTTCAGAAGTACAGAGACTCGCAAGGGTTCAAGGACTCCCTTACCGACGACGTGAAAGGACTGCTGAGCTTGTACGAAGCTTCTTTCCTTTCATTCCCAGGAGAACAACTGATGGAGGAAGCTAATAAGTTCAGCGTTAGGCACCTAGAGAGTTTGACTCAAAAGGTAGGGTTAGATATCGAGGAACAAGTGAGACACTCTCTACAGGTTCCATTGCACCGTAGGATGAGGAGACTCGAAGCTAGGGAGTACATAGATGTGTACCAGAGGGAGGAGGGGAAGAGCTCAGTCCTGCTAGAGTTTGCCAAGGTCGATTTCAACTTTGTGCAAATCATACATCAAATGGAATTGAAAGAACTCTCCAAATGGTGGATAAGCTTGAATTTGGACAGTGTGCTCAGTTTCACAAGAGACAGATTAGTTGAAAATTATCTATGGGCTATCGGATTAGTCTATGAGCCCCATATGTCAAAATGTAGGATCGGCATCACTAAGGCAGTATGCATTTTATCCGTTATTGATGATGTCTATGATATTTATGGGTCTTTTGAGGAAGCGGAGATCTTAACAAAGACGATCAAAAGTTGGGATCCTCATGAAATGAGAAACCTCCCCGAAAATATCAAGTTATGCTATAAGATAATTTACAATTTTATTGAGGAGATTACCACATCTATTCTATTGGATTATGGGTGCAATGTTATGCCCTTTCTAAAAAAAGAGTGGGAGAATTTATGTGGAGCATATTTGGTAGAAGCAAAATGGTTCCATGAAGGCTATACTCCTTCTCTTAAAGAATACCTAAAGAATGCGTGGATTTCAATTGGTGGGCCGATGACCTTTGTTTTTGCTTATTGTCTCCTAGGACACACATTGGGAGATAATTCCCTTAATTGCTTAAAACAAGGTTTCGATCCTATATATTGGTCTTCACTAATACTCCGTCTCAATGATGATTTGGGAACTTCTAAGGCCGAAATGGAAAGAGGAGACACACCCAAATCAATTCAATGCCATATGAGAGAAACATGTGAATTAGAAGAGGTATCTAGAGAATACATAAAGAACTTAGTGGATCATTTTTGGAAGGAGTTGAATCAAAAATCTATAAGAACTCATCTTCCGAAGAATTTTATGAACTTAGTCACAAACATGGCCCTAGCATCTCACTGCATTTTCCAATTTGGAGATGGAATTGGAGATTCCACTGGCATAACAAAAAATCGTATCCTCTCACTATTTGTCAATAATGTCCCATTGGAATGA

>LpdTPS-1,MH203233

ATGGCAGCTATGAGCTGTTTCTCACTGGCCCGACTTCCCATCTCATCCACTTCTTCGAGTACTCGCAATTTCAGTTGTCTAACAAAACCTCATTCAATGCGGAGGTCATGCAAGATAGTCAATACTACGGAGAAATTGGAAGAACCTCCCCGTCGATCCGCCAATTACCACCCAACCATATGGGATCACTCGACCATCCAATCCATTGAGAATTTTAGTTCATTAAAGGGGAACACACTTGAAAGGCGAAGAGAATCACTTAAGAATGAAGTGAAGTTATTGCTTGATGCATCAGACGATCCAGTGGCTCAGCTCCAGCTCATTGACACTATTCAACGGCTTGGCATCGCCTACCATTTCGATAACGAGATTAAATCTATCCTTGATAGAATACGTGATTGTCATTTTGATCTAGAGGCTTTGGACTGTGTCCATAAGACGGCTCTTGCCTTTCGTCTACTCCGACAACATGGTTACGATGTGTCATCAGATGTTTTTCAGAAGTACAGAGACTCGCAAGGGTTCAAGGACTCCCTTACCGACGACGTGAAAGGACTGCTGAGCTTGTACGAAGCTTCTTTCCTTTCATTTCCAGGAGAACAACTGATGGAGGAAGCTAATAAGTTCAGCGTTAGGCACCTAGAGAGTTTGACTCAAAAGGTAGGGTTAGATATCGAGGAACAAGTGAGACACTCTCTACAAGTTCCATTGCATCGGAGGATGAGGAGACTCGAAGCTAGGGAGTACATAGATGTGTACCAGAGAGAGGAGGGGAAGAGCTCAGTCCTGCTAGAGTTTGCCAAGGTCGATTTCAACTTTGTGCAAATCATACATCAAATGGAATTGAAAGAACTCTCCAAATGGTGGATAAGCTTGAATTTGGGTAGTGTGCTCAGTTTTACCAGAGACAGATTAGTTGAAAATTATCTATGGGCTATCGGATTTGTCTATGAGCCCCATATGTCAAAATGTAGGATCGGCATCACTAAGGCGGTATGCATTTTATCCGTTATTGATGATGTCTATGATATATATGGGTCTTTTGAGGAAGTGGAGATCTTAACAAAGACGATCAAAAGTTGGGATCCTCATGAAATGAGAAACCTCCCCGAAAATATCAAGTTATGCTATAAGATACTTTACAATTTTATTGAGGAGATTACCACATGTACTCTATTGGATCATGGGTGCAATGTTATGCCCTTTCTAAAAGAAGAGTGGGAGAATTTATGTGGAGCATTTTTGGTAGAAGCAAAATGGTTCCATGAAGGCTATACTCCTTCTCTTAAAGAATACCTAAAGAATGCGTGGATTTCAATTGGTGGGCCGATGACCTTTGTGTTTGCTTATTGTCTCCTAGGTCACACATTGGGAGATAATTCCCTTAATTGCTTAAAACAAGGTTTCGATCCTATATACTGGTCTTCACTGATACTCCGTCTCAATGATGATTTGGGAACTTCTAAGGTCGAAATGGAAAGAGGAGACACACCCAAACCAATTCAATGCCATATGAGAGAAACAGATGAATCAGATGAGGTATCTAGAGAATACATAAAGAACTTAGTGGACCATTTTTGGAAGGAGTTGAATCAAGAATCTATAAGAACTCATCTTCCAAAGAATTTTATGAACTTAGTCACGAACATGGCCCTAGCATCTCACTGCATCTTCCAATTTGGAGATGGAATTGGAGATTCCACTGGCATAACAAAAAATCGTATCCTCTCACTATTTTTCAATAATGTCCCATTGGAATGA

>LbeTPS-1,MH203242

ATGGCAGCTATGAGCTGTTTCTCACTGGCCCGACTTCCCATCTCATCCACTTCTTCGAGTACTCGCAATTTCAGTTGTCTAACAAAACCTCATTCAATTCAGAGGTCATGCAAGATAGTCAATACTACGGAGAAATTGGAAGAACCTCCCCGTCGATCCGCCAATTACCACCCAACCATATGGGATCACTCGACCATCCAATCCATTGAGAGTTTGAGTTCATTAAAGGGGAACACACTTGAAAGGCGAAGAGAATCACTTAAGAATGAAGTGAAGTTATTGCTTGATGCATCAGACGATCCAGTGGCTCAGCTCCAGCTCATTGACACTATTCAACGGCTTGGCATCGCCTACCATTTCGATAACGAGATTAAATCTATCCTTGATAGAATACGTGATTGTCATTTTGATCTAGAGGCTTTGGACTGTGTCCATAAGACGGCTCTTGCCTTTCGTCTACTCCGACAACATGGTTACGATGTGTCATCAGATGTTTTTCAGAAGTACAGAGACTCGCAAGGGTTCAAGGACTCCCTTACCGACGACGTGAAAGGACTGCTGAGCTTGTACGAAGCTTCTTTCCTTTCATTTCCAGGAGAACAACTGATGGAGGAAGCTAATAAGTTCAGCGTTAGGCACCTAGAGAGTTTGACTCAAAAGGTAGGGTTAGATATCGAGGAACAAGTGAGACACTCTCTACAAGTTCCATTGCATCGGAGGATGAGGAGACTCGAAGCTAGGGAGTACATAGATGTGTACCAGAGAGAGGAGGGGAAGAGCTCAGTCCTGCTAGAGTTTGCCAAGGTCGATTTCAACTTTGTGCAAATCATACATCAAATGGAATTGAAAGAACTCTCCAAATGGTGGATAAGCTTGAATTTGGGTAGTGTGCTCAGTTTTACCAGAGACAGATTAGTTGAAAATTATCTATGGGCTATCGGATTTGTCTATGAGCCCCATATGTCAAAATGTAGGATCGGCATCACTAAGGCGGTATGCATTTTATCCGTTATTGATGATGTCTATGATATATATGGGTCTTTTGAGGAAGTGGAGATCTTAACAAAGACGATCAAAAGTTGGGATCCTCATGAAATGAGAAACCTCCCCGAAAATATCAAGTTATGCTATAAGATACTTTACAATTTTATTGAGGAGATTACCACATGTACTCTATTGGATCATGGGTGCAATGTTATGCCCTTTCTAAAAGAAGAGTGGGAGAATTTATGTGGAGCATTTTTGGTAGAAGCAAAATGGTTCCATGAAGGCTATACTCCTTCTCTTAAAGAATACCTAAAGAATGCGTGGATTTCAATTGGTGGGCCGATGACCTTTGTGTTTGCTTATTGTCTCCTAGGTCACACATTGGGAGATAATTCCCTTAATTGCTTAAAACAAGGTTTCGATCCTATATACTGGTCTTCACTGATACTCCGTCTCAATGATGATTTGGGAACTTCTAAGGTCGAAATGGAAAGAGGAGACACACCCAAATCAATTCAATGCCATATGAGAGAAACAGATGAATCAGATGAGGTATCTAGAGAATACATAAAGAACTTAGTGGACCATTTTTGGAAGGAGTTGAATCAAGAATCTATAAGAACTCATCTTCCAAAGAATTTTATGAACTTAGTCACGAACATGGCCCTAGCATCTCACTGCATCTTCCAATTTGGAGATGGAATTGGAGATTCCACTGGCATAACAAAAAATCGTATCCTCTCACTATTTTTCAATAATGTCCCATTGGAATGA

>LzaTPS-1,MH203255

ATGGCAGCTATGAGCTGTTTCTCACTGGCCCGACTTCCCATCTCATCCACTTCTTCGAGTACTCGCAATTTCAGTTGTCTAACAAAACCTCATTCAATGCGGAGGTCATGCAAGATAGTCAATACTACGGAGAAATTGGAAGAACCTCCCCGTCGATCCGCCAATTACCACCCAACCATATGGGATCACTCGACCATCCAATCCATTGAGAATTTTAGTTCATTAAAGGGGAACACACTTGAAAGGCGAAGAGAATCACTTAAGAATGAAGTGAAGTTATTGCTTGATGCATCAGACGATCCAGTGGCTCAGCTCCAGCTCATTGACACTATTCAACGGCTTGGCATCGCCTACCATTTCGATAACGAGATTAAATCTATCCTTGATAGAATACGTGATTGTCATTTTGATCTAGAGGCTTTGGACTGTGTCCATAAGACGGCTCTTGTCTTTCGTCTACCCCGACAACATGGTTACGATGTGTCATCAGATGTTTTTCAGAAGTACAGAGACTCGCAAGGGTTCAAGGACTCCCTTACCGACGACGTGAAAGGACTGCTGAGCTTGTACGAAGCTTCTTTCCTTTCATTTCCAGGAGAACAACTGATGGAGGAAGCTAATAAGTTCAGCGTTAGGCACCTAGAGAGTTTGACTCAAAAGGTAGGGTTAGATATCGAGGAACAAGTGAGACACTCTCTACAAGTTCCATTGCATCGGAGGATGAGGAGACTCGAAGCTAGGGAGTACATAGATGTGTACCAGAGAGAGGAGGGGAAGAGCTCAGTCCTGCTAGAGTTTGCCAAGGTCGATTTCAACTTTGTGCAAATCATACATCAAATGGAATTGAAAGAACTCTCCAAATGGTGGATAAGCTTGAATTTGGGTAGTGTGCTCAGTTTTACCAGAGACAGATTAGTTGAAAATTATCTATGGGCTATCGGATTTGTCTATGAGCCCCATATGTCAAAATGTAGGATCGGCATCACTAAGGCGGTATGCATTTTATCCGTTATTGATGATGTCTATGATATATATGGGTCTTTTGAGGAAGTGGAGATCTTAACAAAGACGATCAAAAGTTGGGATCCTCATGAAATGAGAAACCTCCCCGAAAATATCAAGTTATGCTATAAGATACTTTACAATTTTATTGAGGAGATTACCACATGTACTCTATTGGATCATGGGTGCAATGTTATGCCCTTTCTAAAAGAAGAGTGGGAGAATTTATGTGGAGCATTTTTGGTAGAAGCAAAATGGTTCCATGAAGGCTATACTCCTTCTCTTAAAGAATACCTAAAGAATGCGTGGATTTCAATTGGTGGGCCGATGACCTTTGTGTTTGCTTATTGTCTCCTAGGTCACACATTGGGAGATAATTCCCTTAATTGCTTAAAACAAGGTTTCGATCCTATATACCGGTCTTCACTGATACTCCGTCTCAATGATGATTTGGGAACTTCTAAGGTCGAAATGGAAAGAGGAGACACACCCAAATCAATTCAATGCCATATGAGAGAAACAGATGAATCAGATGAGGTATCTAGAGAATACATAAAGAACTTAGTGGACCATTTTTGGAAGGAGTTGAATCAAGAATCTATAAGAACTCATCTTCCAAAGAATTTTATGAACTTAGTCACGAACATGGCCCTAGCATCTCACTGCATCTTCCAATTTGGAGATGGAATTGGAGATTCCACTGGCATAACAAAAAATCGTATCCTCTCACTATTTTTCAATAATGTCCCATTGGAATGA

>LfrTPS-1,MH203256

ATGGCAGCTATGAGCTGTTTCTCACTGGCCCGACTTCCCATCTCATCCACTTCTTCGAGTACTCGCAATTTCAGTTGTCTAACAAAACCTCATTCAATGCGGAGGTCATGCAAGATAGTCAATACTACGGAGAAATTGGAAGAACCTCCCCGTCGATCCGCCAATTACCACCCAACCATATGGGATCACTCGACCATCCAATCCATTGAGAATTTTAGTTCATTAAAGGGGAACACACTTGAAAGGCGAAGAGAATCACTTAAGAATGAAGTGAAGTTATTGCTTGATGCATCAGACGATCCAGTGGCTCAGCTCCAGCTCATTGACACTATTCAACGGCTTGGCATCGCCTACCATTTCGATAACGAGATTAAATCTATCCTTGATAGAATACGTGATTGTCATTTTGATCTAGAGGCTTTGGACTGTGTCCATAAGACGGCTCTTGCCTTTCGTCTACTCCGACAACATGGTTACGATGTGTCATCAGATGTTTTTCAGAAGTACAGAGACTCGCAAGGGTTCAAGGACTCCCTTACCGACGACGTGAAAGGACTGCTGAGCTTGTACGAAGCTTCTTTCCTTTCATTTCCAGGGGAACAACTGATGGAGGAAGCTAATAAGTTCAGCGTTAGGCACCTAGAGAGTTTGACTCAAAAGGTAGGGTTAGATATCGAGGAACAAGTGAGACACTCTCTACAAGTTCCATTGCATCGGAGGATGAGGAGACTCGAAGCTATGGAGTACATAGATGTGTACCAGAGAGAGGAGGGGAAGAGCTCAGTCCTGCTAGAGTTTGCCAAGGTCGATTTCAACTTTGTGCAAATCATACATCAAATGGAATTGAAAGAACTCTCCAAATGGTTGATGAGCTTGAATTTGGGTAGTGTGCTCAGTTTTACCAGGGACAGATTAGTTGAAAATTATCTATGGGCTATCGGATTTGTCTATGAGCCCCATATGTCAAAATGTAGGATCGGCATCACTAAGGCGGTATGCATTTTATCCGTTATTGATGATGTCTATGATATATATGGGTCTTTTGAGGAAGTGGAGATCTTAACAAAGACGATCAAAAGTTGGGATCCTCATGAAATGAGAAACCTCCCCGAAAATATCAAGTTATGCTATAAGATACTTTACAATTTTATTGAGGAGATTACCACATGTACTCTATTGGATCATGGGTGCAATGTTATGCCCTTTCTAAAAGAAGAGTGGGAGAATTTATGTGGAGCATTTTTGGTAGAAGCAAAATGGTTCCATGAAGGCTATACTCCTTCTCTTAAAGAATACCTAAAGAATGCGTGGATTTCAATTGGTGGGCCGATGACCTTTGTGTTTGCTTATTGTCTCCTAGGTCACACATTGGGAGATAATTCCCTTAATTGCTTAAAACAAGGTTTCGATCCTATATACTGGTCTTCACTGATACTCCGTCTCAATGATGATTTGGGAACTTCTAAGGTCGAAATGGAAAGAGGAGACACACCCAAATCAATTCAATGCCATATGAGAGAAACAGATGAATCAGATGAGGTATCTAGAGAATACATAAAGAACTTAGTGGACCATTTTTGGAAGGAGTTGAATCAAGAATCTATAAGAACTCATCTTCCAAAGAATTTTATGAACTTAGTCACGAACATGGCCCTAGCATCTCACTGCATCTTCCAATTTGGAGATGGAATTGGAGATTCCACTAGCATAACAAAAAATCGTATCCTCTCACTATTTTTCAATAATGTCCCATTGGAATGA

>LurTPS-1,MH203257

ATGGCAGCTATGAGCTGTTTCTCACTGGCCCGACTTCCCATCTCATCCACTTCTTCGAGTACTCGCAATTTCAGTTGTCTAACAAAACCTCATTCAATGCGGAGGTCATGCAAGATAGTCAATACTACGGAGAAATTGGAAGAACCTCCCCGTCGATCCGCCAATTACCACCCAACCATATGGGATCACTCGACCATCCAATCCATTGAGAATTTTAGTTCATTAAAGGGGAACACACTTGAAAGGCGAAGAGAATCACTTAAGAATGAAGTGAAGTTATTGCTTGATGCATCAGACGATCCAGTGGCTCAGCTCCAGCTCATTGACACTATTCAACGGCTTGGCATCGCCTACCATTTCGATAACGAGATTAAATCTATCCTTGATAGAATACGTGATTGTCATTTTGATCTAGAGACTTTGGACTGTGTCCATAAGACGGCTCTTGCCTTTCGTCTACTCCGACAACATGGTTACGATGTGTCATCAGATGTTTTTCAGAAGTACAGAGACTCGCAAGGGTTCAAGGACTCCCTTACCGACGACGTGAAAGGACTGCTGAGCTTGTACGAAGCTTCTTTCCTTTCATTTCCAGGAGAACAACTGATGGAGGAAGCTAATAAGTTCAGCGTTAGGCACCTAGAGAGTTTGACTCAAAAGGTAGGGTTAGATATCGAGGAACAAGTGAGACACTCTCTACAAGTTCCATTGCATCGGAGGATGAGGAGACTCGAAGCTAGGGAGTACATAGATGTGTACCAGAGAGAGGAGGGGAAGAGCTCAGTCCTGCTAGAGTTTGCCAAGGTCGATTTCAACTTTGTGCAAATCATACATCAAATGGAATTGAAAGAACTCTCCAAATGGTTGATAAGCTTGAATTTGGGTAGTGTGCTCAGTTTTACCAGGGACAGATTAGTTGAAAATTATCTATGGGCTATCGGATTTGTCTATGAGCCCCATATGTCAAAATGTAGGATCGGCATCACTAAGGCGGTATGCATTTTATCCGTTATTGATGATGTCTATGATATATATGGGTCTTTTGAGGAAGTGGAGATCTTAACAAAGACGATCAAAAGTTGGGATCCTCATGAAATGAGAAACCTCCCCGAAAATATCAAGTTATGCTATAAGATACTTTACAATTTTATTGAGGAGATTACCACATGTACTCTATTGGATCATGGGTGCAATGTTATGCCCTTTCTAAAAGAAGAGTGGGAGAATTTATGTGGAGCATTTTTGGTAGAAGCAAAATGGTTCCATGAAGGCTATACTCCTTCTCTTAAAGAATACCTAAAGAATGCGTGGATTTCAATTGGTGGGCCGATGACCTTTGTGTTTGCTTATTGTCTCCTAGGTCACACATTGGGAGATAATTCCCTTAATTGCTTAAAACAAGGTTTCGATCCTATATACTGGTCTTCACTGATACTCCGTCTCAATGATGATTTGGGAACTTCTAAGGTCGAAATGGAAAGAGGAGACACACCCAAATCAATTCAATGCCATATGAGAGAAACAGATGAATCAGATGAGGTATCTAGAGAATACATAAAGAACTTAGTGGACCATTTTTGGAAGGAGTTGAATCAAGAATCTATAAGAACTCATCTTCCAAAGAATTTTATGAACTTAGTCACGAACATGGCCCTAGCATCTCACTGCATCTTCCAATTTGGAGATGGAATTGGAGATTCCACTAGCATAACAAAAAATCGTATCCTCTCACTATTTTTCAATAATGTCCCATTGGAATGA

>LroTPS-1,MH203258

ATGGCAGCTATGAGCTGTTTCTCACTGGCCCGACTTCCCATCTCATCCACTTCTTCGAGTACTCGCAATTTCAGTTGTCTAACAAACCCTCATTCAATGCGGAGGTCATGCAAGATAGTCAATACTACGGAGAAATTGGAAGAACCTCCCCGTCGATCCGCCAATTACCACCCAACCATATGGGATCACTCGACCATCCAATCCATTGAGAATTTTAGTTCATTAAAGGGGAACACACTTGAAAGGCGAAGAGAATCACTTAAGAATGAAGTGAAGTTATTGCTTGATGCATCAGACGATCCAGTGGCTCAGCTCCAGCTCATTGACACTATTCAACGGCTTGGCATCGCCTACCATTTCGATAACGAGATTAAATCTATCCTTGATAGAATACGTGATTGTCATTTTGATCTAGAGGCTTTGGACTGTGTCCATAAGACGGCTCTTGCCTTTCGTCTACTCCGACAACGTGGTTACGATGTGTCATCAGATGTTTTTCAGAAGTACAGAGACTCGCAAGGGTTCAAGGACTCCCTTACCGACGACGTGAAAGGACCGCTGAGCTTGTACGAAGCTTCTTTCCTTTCATTTCCAGGAGAACAACTGATGGAGGAAGCTAATAAGTTCAGCGTTAAGCACCTAGAGAGTTTGACTCAAAAGGTAGGGTTAGATATCGAGGAACAAGTGAGACACTCTCTACAAGTTCCATTGCGTCGGAGGATGAGGAGACTCGAAGCTAGGGAGTACATAGATGTGTACCAGAGAGAGGAGGGGAAGAGCTCAGTCCTGCTAGAGTTTGCCAAGGTCGATTTCAACTTTGTGCAAATCATACATCAAATGGAATTGAAAGAACTCTCCAAATGGTGGATAAGCTTGAATTTGGGTAGTGTGCTCAGTTTTACCAGAGACAGATTAGTTGAAAATTATCTATGGGCTATCGGATTTGTCTATGAGCCCCATATGTCAAAATGTAGGATCGGCATCACTAAGGCGGTATGCATTTTATCCGTTATTGATGATGTCTATGATATATACGGGTCTTTTGAGGAAGTGGAGATCTTAACAAAGACGATCAAAAGTTGGGATCCTCATGAAATGAGAAACCTCCCCGAAAATATCAAGTTATGCTATAAGATACTTTACAATTTTATTGAGGAGATTACCACATGTACTCTATTGGATCATGGGTGCAATGTTATGCCCTTTCTAAAAGAAGAGTGGGAGAATTTATGTGGAGCATTTTTGGTAGAAGCAAAATGGTTCCATGAAGGCTATACTCCTTCTCTTAAAGAATACCTAAAGAATGCGTGGATTTCAATTGGTGGGCCGATGACCTTTGTGTTTGCTTATTGTCTCCTAGGTCACACATTGGGAGATAATTCCCTTAATTGCTTAAAACAAGGTTTCGATCCTATATACTGGTCTTCACTGATACTCCGTCTCAATGATGATTTGGGAACTTCTAAGGTCGAAATGGAAAGAGGAGACACACCCAAATCAATTCAATGCCATATGAGAGAAACAGATGAATCCGATGAGGTATCTAGAGAATACATAAAGAACTTAGTGGACCATTTTTGGAAGGAGTTGAATCAAGAATCTATAAGAACTCATCTTCCAAAGAATTTTATGAACTTAGTCACGAACATGGCCCTAGCATCTCACTGCATCTTCCAATTTGGAGATGGAATTGGAGATTCCACTGGCATAACAAAAAATCGTATCCTCTCACTATTTTTCAATAATGTCCCATTGGAATGA

>LmsTPS-1,MH203265

ATGGCAGCTATGAGCTGTTTCTCACTGGCCCGACTTCCCATCTCATCCACTTCTTCGAGTACTCGCAATTTCAGTTGTCTAACAAAACCTCATTCAATGCGGAGGTCATGCAAGATAGTCAATACTACGGAGAAATTGGAAGAACCTCCCCGTCGATCCGCCAATTACCACCCAACCATATGGGATCACTCGACCATCCAATCCATTGAGAATTTTAGTTCATTAAAGGGGAACACACTTGAAAGGCGAAGAGAATCACTTAAGAATGAAGTGAAGTTATTGCTTGATGCATCAGACGATCCAGTGGCTCAGCTCCAGCTCATTGACACTATTCAACGGCTTGGCATCGCCTACCATTTCGATAACGAGATTAAATCTATCCTTGATAGAATACGTGATTGTCATTTTGATCTAGAGGCTTTGGACTGTGTCCATAAGACGGCTCTTGCCTTTCGTCTACTCCGACAACATGGTTACGATGTGTCATCAGATGTTTTTCAGAAGTACAGAGACTCGCAAGGGTTCAAGGACTCCCTTACCGACGACGTGAAAGGACTGCTGAGCTTGTACGAAGCTTCTTTCCTTTCATTTCCAGGAGAACAACTGATGGAGGAAGCTAATAAGTTCAGCGTTAGGCACCTAGAGAGTTTGACTCAAAAGGTAGGGTTAGATATCGAGGAACAAGTGAGACACTCTCTACAAGTTCCATTGCATCGGAGGATGAGGAGACTCGAAGCTAGGGAGTACATAGATGTGTACCAGAGAGAGGAGGGGAAGAGCTCAGTCCTGCTAGAGTTTGCCAAGGTCGATTTCAACTTTGTGCAAATCATACATCAAATGGAATTGAAAGAACTCTCCAAATGGTGGATAAGCTTGAATTTGGGTAGTGTGCTCAGTTTTACCAGAGACAGATTAGTTGAAAATTATCTATGGGCTATCGGATTTGTCTATGAGCCCCATATGTCAAAATGTAGGATCGGCATCACTAAGGCGGTATGCATTTTATCCGTTATTGATGATGTCTATGATATATATGGGTCTTTTGAGGAAGTGGAGATCTTAACAAAGACGATCAAAAGTTGGGATCCTCATGAAATGAGAAACCTCCCCGAAAATATCAAGTTATGCTATAAGATACTTTACAATTTTATTGAGGAGATTACCACATGTACTCTATTGGATCATGGGTGCAATGTTATGCCCTTTCTAAAAGAAGAGTGGGAGAATTTATGTGGAGCATTTTTGGTAGAAGCAAAATGGTTCCATGAAGGCTATACTCCTTCTCCTAAAGAATACCTAAAGAATGCGTGGATTTCAATTGGTGGGCCGATGACCTTTGTGTTTGCTTATTGTCTCCTAGGTCACACATTGGGAGATAATTCCCTTAATTGCTTAAAACAAGGTTTCGATCCTATATACTGGTCTTCACTGATACTCCGTCTCAATGATGATTTGGGAACTTCTAAGGTCGAAATGGAAAGAGGAGACACACCCAAACCAATTCAATGCCATATGAGAGAAACAGATGAATCAGATGAGGTATCTAGAGAATACATAAAGAACTTAGTGGACCATTTTTGGAAGGAGTTGAATCAAGAATCTATAAGAACTCATCTTCCAAAGAATTTTATGAACTTAGTCACGAACATGGCCCTAGCATCTCACTGCATCTTCCAATTTGGAGATGGAATTGGAGATTCCACTGGCATAACAAAAAATCGTATCCTCTCACTATTTTTCAATAATGTCCCATTGGAATGA

>LgsTPS-1,MH203261

ATGGCAGCTATGAGCTGTTTCTCACTGGCCCGACTTCCCATCTCATCCACTTCTTCGAGTACTCGCAATTTCAGTTGTCTAACAAAACCTCATTCAATGCGGAGGTCATGCAAGATAGTCAATACTACGGAGAAATTGGAAGAACCTCCCCGTCGATCCGCCAATTACCACCCAACCATATGGGATCACTCGACCATCCAATCCATTGAGAATTTTAGTTCATTAAAGGGGAACACACTTGAAAGGCGAAGAGAATCACTTAAGAATGAAGTGAAGTTATTGCTTGATGCATCAGACGATCCAGTGGCTCAGCTCCAGCTCATTGACACTATTCAACGGCTTGGCATCGCCTACCATTTCGATAACGAGATTAAATCTATCCTTGATAGAATACGTGATTGTCATTTTGATCTAGAGGCTTTGGACTGTGTCCATAAGACGGCTCTTGCCTTTCGTCTACTCCGACAACATGGTTACGATGTGTCATCAGATGTTTTTCAGAAGTACAGAGACTCGCAAGGGTTCAAGGACTCCCTTACCGACGACGTGAAAGGACTGCTGAGCTTGTACGAAGCTTCTTTCCTTTCATTTCCAGGAGAACAACTGATGGAGGAAGCTAATAAGTTCAGCGTTAGGCACCTAGAGAGTTTGACTCAAAAGGTAGGGTTAGATATCGAGGAACAAGTGAGACACTCTCTACAAGTTCCATTGCATCGGAGGATGAGGAGACTCGAAGCTAGGGAGTACATAGATGTGTACCAGAGAGAGGAGGGGAAGAGCTCAGTCCTGCTAGAGTTTGCCAAGGTCGATTTCAACTTTGTGCAAATCATACATCAAATGGAATTGAAAGAACTCTCCAAATGGTGGATAAGCTTGAATTTGGGTAGTGTGCTCAGTTTTACCAGAGACAGATTAGTTGAAAATTATCTATGGGCTATCGGATTTGTCTATGAGCCCCATATGTCAAAATGTAGGATCGGCATCACTAAGGCGGTATGCATTTTATCCGTTATTGATGATGTCTATGATATATATGGGTCTTTTGAGGAAGTGGAGATCTTAACAAAGACGATCAAAAGTTGGGATCCTCATGAAATGAGAAACCTCCCCGAAAATATCAAGTTATGCTATAAGATACTTTACAATTTTATTGAGGAGATTACCACATGTACTCTATTGGATCATGGGTGCAATGTTATGCCCTTTCTAAAAGAAGAGTGGGAGAATTTATGTGGAGCATTTTTGGTAGAAGCAAAATGGTTCCATGAAGGCTATACTCCTTCTCTTAAAGAATACCTAAAGAATGCGTGGATTTCAATTGGTGGGCCGATGACCTTTGTGTTTGCTTATTGTCTCCTAGGTCACACATTGGGAGATAATTCCCTTAATTGCTTAAAACAAGGTTTCGATCCTATATACTGGTCTTCACTGATACTCCGTCTCAATGATGATTTGGGAACTTCTAAGGTCGAAATGGAAAGAGGAGACACACCCAAATCAATTCAATGCCATATGAGAGAAACAGATGAATCAGATGAGGTATCTAGAGAATACATAAAGAACTTAGTGGACCATTTTTGGAAGGAGTTGAATCAAGAATCTATAAGAACTCATCTTCCAAAGAATTTTATGAACTTAGTCACGAACATGGCCCTAGCATCTCACTGCATCTTCCAATTTGGAGATGGAATTGGAGATTCCACTGGCATAACAAAAAATCGTACCCTCTCACTATTTTTCAATAATGTCCCATTGGAATGA

>LamTPS-1,MH203283

ATGGCAGCTATGAGCTGTTTCTCACTGGCCCGACTTCCCATCTCATCCACTTCTTCGAGTACTCGCAATTTCAGTTGTCTAACAAAACCTCATTCAATTCAGAGGTCATGCAAGATAGTCAATACTACGGAGAAATTGGAAGAACCTCCCCGTCGATCCGCCAATTACCACCCAACCATATGGGATCACTCGACCATCCAATCCATTGAGAGTTTGAGTTCATTAAAGGGGAACACACTTGAAAGGCGAAGAGAATTACTTAAGAATGAAGTGAAGTTATTGCTTGATGCATCAGACGATCCATTGGCTCAGCTCCAGCTCATTGACACTATTCAACGGCTTGGCATCGCCTACCATTTCGATAACGAGATTAAATCTATCCTTGATAGAATACATGATTGTCATGTTCATCTAGAGGCTTTGGACTGTGTCCATAAGACGGCTCTTGCCTTTCGTCTACTCCGACAACATGGTTACGATGTGTCATCAGATGTTTTTCAGAAGTACAGAGACTCGCAAGGGTTCAAGGACTCCCTTACCGACGACGTGAAAGGACTGCTGAGCTTGTACGAAGCTTCTTTCCTTTCATTCCCAGGAGAACAACTGATGGAGGAAGCTAATAAGTTCAGCGTTAGGCACCTAGAGAGTTTGACTCAAAAGGTAGGGTTAGATATCGAGGAACAAGTGAGACACTCTCTACAAGTTCCATTGCACCGGAGGATGAGGAGACTCGAAGCTAGGGAGTACATAGATGTGTACCAGAGGGAGGAGGGGAAGAGCTCAGTCCTGCTAGAGTTTGCCAAGGTCGATTTCAACTTTGTGCAAATCATACATCAAATGGAATTGAAAGAACTCTCCAAATGGTGGATAAGCTTGAATTTGGACAGTGTGCTCAGTTTCATAAGAGACAGATTAGTTGAAAATTATCTATGGGCTATCGGATTTGTCTATGAGCCCCATATGTCAAAATGTAGGATCGGCATCACTAAGGCGGTATGCATTTTATCCGTTATTGATGATGTCTATGATATTTATGGGTCTTTTGAGGAAGTGGAGATCTTAACAAAGACGATCAAAAGTTGGGATCCTCATGAAATGAGAAACCTCCCCGAAAATATCAAGTTATGCTATAAGATAATTTACAGTTTTATTGAGGAGATTACCACATCTATTCTATTGGATAATGGGTGCAATGTTATGCCCTTTCTAAAAAAAGAGTGGGAGAATTTATGTGGATCATATTTGGTAGAAGCAAAATGGTTCCATGAAGGCTATACTCCTTCTCTTAAAGAATACCTAAAGAATGCGTGGATTTCAATTGGTGGGCCGATGACCTTTGTTTTTGCTTATTGTCTCCTAGGACACACATTGGGAGATAATTCCCTTAATTGCTTAAAACAAGGTTTCGATCCTATATACTGGTCTTCACTAATACTCCGTCTCAATGATGATTTGGGAACTTCTAAGGCCGAAATGGAAAGAGGAGACACGCCCAAATCAATTCAATGCCATATGAGAGAAACAGGTGAATCAGAAGAGGTATCTAGAGAATACATAAAGAACTTAGTGGACCATTTTTGGAAGGAGTTGAATCAAGAATCTATAAGAACTCATCTTCCGAAGAATTTTATGAACTTAGTCACGAACATGGCCCTAGCATCTCACTGCATTTTCCAATTTGGAGATGGAATTGGAGATTCCACTGGCATAACAAAAAATCGTATCCTCTCACTATTTGTCAATAATGTCCCATTGGAATGA

>LfoTPS-1,MH203287

ATGGCAGCTATGAGCTGTTTCTCACTGGCCCGACTTCCCATCTCATCCACTTCTTCGAGTACTCGCAATTTCAGTTGTCTAACAAAACCTCATTCAATTCAGAGGTCATGCAAGATAGTCAATACTACGGAGAAATTGGAAGAACCTCCCCGTCGATCCGCCAATTACCACCCAACCATATGGGATCACTCGACCATCCAATCCATTGAGAGTTTGAGTTCATTAAAGGGGAACACACTTGAAAGGCGAAGAGAATTACTTAAGAATGAAGTGAAGTTATTGCTTGATGCATCAGACGATCCAGTGGCTCAGCTCCAGCTCATTGACACTATTCAACGGCTTGGCATCGCCTACCATTTCGATAACGAGATTAAATCTATCCTTGATAGAATACATGATTGTCATGTTCATCTAGAGGCTTTGGACTGTGTCCATAAGACGGCTCTTGCCTTTCGTCTACTCCGACAACATGGTTACGATGTGTCATCAGATGTTTTTCAGAAGTACAGAGACTCGCAAGGGTTCAAGGACTCCCTTACCGACGACGTGAAAGGACTGCTGAGCTTGTACGAAGCTTCTTTCCTTTCATTCCCAGGAGAACAACTGATGGAGGAAGCTAATAAGTTCAGCGTTAGGCACCTAGAGAGTTTGACTCAAAAGGTAGGGTTAGATATCGAGGAACAAGTGAGACACTCTCTACAAGTTCCATTGCACCGGAGGATGAGGAGACTCGAAGCTAGGGAGTACATAGATGTGTACCAGAGGGAGGAGGGGAAGAGCTCAGTCCTGCTAGAGTTTGCCAAGGTCGATTTCAACTTTGTGCAAATCATACATCAAATGGAATTGAAAGAACTCTCCAAATGGTGGATAAGCTTGAATTTGGACAGTGTGCTCAGTTTCATAAGAGACAGATTAGTTGAAAATTATCTATGGGCTATCGGATTTGTCTATGAGCCCCATATGTCAAAATGTAGGATCGGCATCACTAAGGCGGTATGCATTTTATCCGTTATTGATGATGTCTATGATATTTATGGGTCTTTTGAGGAAGTGGAGATCTTAACAAAGACGATCAAAAGTTGGGATCCTCATGAAATGAGAAACCTCCCCGAAAATATCAAGTTATGCTATAAGATAATTTACAATTTTATTGAGGAGATTACCACATCTATTCTATTGGATAATGGGTGCAATGTTATGCCCTTTCTAAAAAAAGAGTGGGAGAATTTATGTGGATCATATTTGGTAGAAGCAAAATGGTTCCATGAAGGCTATACTCCTTCTCTTAAAGAATACCTAAAGAATGCGTGGATTTCAATTGGTGGGCCGATGACCTTTGTTTTTGCTTATTGTCTCCTAGGACACACATTGGGAGATAATTCCCTTAATTGCTTAAAACAAGGTTTCGATCCTATATACTGGTCTTCACTAATACTCCGTCTCAATGATGATTTGGGAACTTCTAAGGCCGAAATGGAAAGAGGAGACACACCCAAATCAATTCAATGCCATATGAGAGAAACAGGTGAATCAGAAGAGGTATCTAGAGAATACATAAAGAACTTAGTGGACCATTTTTGGAAGGAGTTGAATCAAGAATCTATAAGAACTCATCTTCCGAAGAATTTTATGAACTTAGTCACGAACATGGCCCTAGCATCTCACTGCATTTTCCAATTTGGAGATGGAATTGGAGATTCCACTGGCATAACAAAAAATCGTATCCTCTCACTATTTGTCAATAATGTCCCATTGGAATGA

>LjuTPS-a,MH203263

ATGGCAGCTATGAGCTGTCTCTCACTGGCCCGACTTCCCATCTCATCCACTTCTTCGAGTACTCGCAATTTCAGTTGTCTAACAAAACCTCATTCAATGCGGAGGTCATGCAAGATAGTCAATACTACGGAGAAATTGGAAGAACCTCCCCGTCGATCCGCCAATTACCACCCAACCATATGGGATCACTCGACCATCCAATCCATTGAGAATTTTAGTTCATTAAAGGGGAACACACTTGAAAGGCGAAGAGAATCACTTAAGAATGAAGTGAAGTTATTGCTTGATGCATCAGACGATCCAGTGGCTCAGCTCCAGCTCATTGACACTATTCAACGGCTTGGCATCGCCTACCATTTCGATAACGAGATTAAATCTATACTTGATAGAATACGTGATTGTCATTTTGATCTAGAGGCTTTGGACTGTGTCCATAAGACGGCTCTTGCCTTTCGTCTACTCCGACAACATGGTTACGATGTGTCATCAGATGTTTTTCAGAAGTACAGAGACTCGCAAGGGTTCAAGGACTCCCTTACCGACGACGTGAAAGGACTGCTGAGCTTGTACGAAGCTTCTTTCCTTTCATTTCCAGGAGAACAACTGATGGAGGAAGCTAATAAGTTCAGCGTTAGGCACCTAGAGAGTTTGACTCAAAAGGTAGGGTTAGATATCGAGGAACAAGTGAGACACTCTCTACAAGTTCCATTGCATCGGAGGATGAGGAGACTCGAAGCTAGGGAGTACATAGATGTGTACCAGAGAGAGGAGGGGAAGAGCTCAATCCTGCTAGAGTTTGCCAAGGTCGATTTCAACTTTGTGCAAATCATACATCAAATGGAATTGAAAGAACTCTCCAAATGGTGGATAAGCTTGAATTTGGGTAGTGTGCTCAGTTTTACCAGAGACAGATTAGTTGAAAATTATCTATGGGCTATCGGATTTGTCTATGAGCCCCATATGTCAAAATGTAGGATCGGCATCACTAAGGCGGTATGCATTTTATCCGTTATTGATGATGTCTATGATATATATGGGTCTTTTGAGGAAGTGGAGATCTTAACAAAGACGATCAAAAGTTGGGATCCTCATGAAATGAGAAACCTCCCCGAAAATATCAAGTTATGCTATAAGATACTTTACAATTTTATTGAGGGGATTACCACATGTACTCTATTGGATCATGGGTGCAATGTTATGCCCTTTCTAAAAGAAGAGTGGGAGAATTTATGTGGAGCATTTTTGGTAGAAGCAAAATGGTTCCATGAAGGCTATACTCCTTCTCTTAAAGAATACCTAAAGAATGCGTGGATTTCAATTGGTGGGCCGATGACCTTTGTGTTTGCTTATTGTCTCCTAGGTCACACATTGGGAGATAATTCCCTTAATTGCTTAAAACAAGGTTTCGATCCTATATACTGGTCTTCACTGATACTCCGTCTCAATGATGATTTGGGAACTTCTAAGGTCGAAATGGAAAGAGGAGACACACCCAAATCAATTCAATGCCATATGAGAGAAACAGATGAATCAGATGAGGTATCTAGAGAATACATAAAGAACTTAGTGGACCATTTTTGGAAGGAGTTGAATCAAGAATCTATAAGAACTCATCTTCCAAAGAATTTTATGAACTTAGTCACGAACATGGCCCTAGCATCTCACTGCATCTTCCAATTTGGAGATGAAATTGGAGATTCCACTGGCATAACAAAAAATCGTATCCTCTCACTATTTTCCAATAATGTCCCATTGGAATGA

>LsiTPS-1,MH203254

ATGGCAGCTATGAGCTGTTTCTCACTGGCCCGACTTCCCATCTCATCCACTCCTTCGAGTACTCGCAATTTCAGTTGTCTAACAAAACCTCATTCAATGCGGAGGTCATGCAAGATAGTCAATACTACGGAGAAATTGGAAGAACCTCCCCGTCGATCCGCCAATTACCACCCAACCATATGGGATCACTCGACCATCCAATCCATTGAGAATTTTAGTTCATTAAAGGGGAACACACTTGAAAGGCGAAGAGAATCACTTAAGAATGAAGTGAAGTTATTGCTTGATGCATCAGACGATCCAGTGGCTCAGCTCCAGCTCATTGACACTATTCAACGGCTTGGCATCGCCTACCATTTCGATAACGAGATTAAATCTATCCTTGATAGAATACGTGATTGTCATTTTGATCTAGAGGCTTTGGACTGTGTCCATAAGACGGCTCTTGCCTTTCGTCTACTCCGACAACATGGTTACGATGTGTCATCAGATGTTTTTCAGAAGTACAGAGACTCGCAAGGGTTCAAGGACTCCCTTACCGACGACGTGAAAGGACTGCTGAGCTTGTACGAAGCTTCTTTCCTTTCATTTCCAGGAGAACAACTGATGGAGGAAGCTAATAAGTTCAGCGTTAGGCACCTAGAGAGTTTGACTCAAAAGGTAGGGTTAGATATCGAGGAACAAGTGAGACACTCTCTACAAGTTCCATTGCATCGGAGGATGAGGAGACTCGAAGCTAGGGAGTACATAGATGTGTACCAGAGAGAGGAGGGGAAGAGCTCAGTCCTGCTAGAGTTTGCCAAGGTCGATTTCAACTTTGTGCAAATCATACATCAAATGGAATTGAAAGAACTCTCCAAATGGTGGATAAGCTTGAATTTGGGTAGTGTGCTCAGTTTTACCAGAGACAGATTAGTTGAAAATTATCTATGGGCTATCGGATTTGTCTATGAGCCCCATATGTCAAAATGTAGGATCGGCATCACTAAGGCGGTATGCATTTTATCCGTTATTGATGATGTCTATGATATATATGGGTCTTTTGAGGAAGTGGAGATCTTAACAAAGACGATCAAAAGTTGGGATCCTCATAAAATGAGAAACCTCCCCGAAAATATCAAGTTATGCTATAAGATACTTTACAATTTTATTGAGGAGATTACCACATGTACTCTATTGGATCATGGGTGCAATGTTATGCCCTTTCTAAAAGAAGAGTGGGAGAATTTATGTGGAGCATTTTTGGTAGAAGCAAAATGGTTCCATGAAGGCTATACTCCTTCTCTTAAAGAATACCTAAAGAATGCGTGGATTTCAATTGGTGGGCCGATGACCTTTGTGTTTGCTTATTGTCTCCTAGGTCACACATTGGGAGATAATTCCCCTAATTGCTTAAAACAAGGTTTCGATCCTATATACTGGTCTTCACTGATACTCCGTCTCAATGATGATTTGGGAACTTCTAAGGTCGAAATGGAAAGAGGAGACACACCCAAATCAATTCAATGCCATATGAGAGAAACAGATGAATCAGATGAGGTATCTAGAGAATACATAAAGAACTTAGTGGACCATTTTTGGAAGGAGTTGAATCAAGAATCTATAAGAACTCATCTTCCAAAGAATTTTATGAACTTAGTCATGAACATGGCCCTAGCATCTCACTGCATCTTCCAATTTGGAGATGGAATTGGAGATTCCACTGGCATAACAAAAAATCGTATCCTCTCACTATTTTTCAATAATGTCCCATTGGAATGA

>LsoTPS-2,MH203303

ATGGCAGCTATGAGCTGTCTCTCACTGGCCCGACTTCCCATCTCATCCACTTCTTCGAGTACTCGCAATTTCAGTTGTCTAACAAAACCTCATTCAATGCGGAGGTCATGCAAGATAGTCAATACTACGGAGAAATCGGAAGAACCTCCCCGTCGATCCGCCAATTACCACCCAACCATATGGGATCACTCGACCATCCAATCCATTGAGAATTTTAGTTCATTAAAGGTAATGTTCCAGGGGAACACACTTGAAAGGCGAAGAGAATCACTTAAGAATGAAGTGAAGTTATTGCTTGATGCATCAGACGATCCAGTGGCTCAGCTCCAGCTCATTGACACTATTCAACGGCTTGGCATCGCCTACCGTTTCGATAACGAGATTAAATCTATCCTTGATAGAATACGTGATTGTCATTTTGATCTAGAGGCTTTGGACTGTGTCCATAAGACGGCTCTTGCCTTTCGTCTACTCCGACAACATGGTTACGATGTGTCATCAGATGTTTTTCAGAAGTACAGAGACTCGCAAGGGTTCAAGGACTCCCTTACCGACGACGTGAAAGGACTGCTGAGCTTGTACGAAGCTTCTTTCCTTTCATTTCCAGGAGAACAACTGATGGAGGAAGCTAATAAGTTCAGCGTTAGGCACCTAGAGAGTTTGACTCAAAAGGTAGGGTTAGATATCGAGGAACAAGTGAGACACTCTCTACAAGTTCCATTGCATCGGAGGATGAGGAGACTCGAAGCTAGGGAGTACATAGATGTGTACCAGAGAGAGGAGGGGAAGAGCTCAGTCCTGCTAGAGTTTGCCAAGGTCGATTTCAACTTTGTGCAAATCATACATCAAATGGAATTGAAAGAACTCTCCAAATGGTGGATAAGCTTGAATTTGGGTAGTGTGCTCAGTTTTACCAGAGACAGATTAGTTGAAAATTATCTATGGGCTATCGGATTCGTCTATGAGCCCCATATGTCAAAATGTAGGATCGGCATCACTAAGGCGGTATGCATTTTATCCGTTATTGATGATGTCTATGATATATATGGGTCTTTTGAGGAAGTGGAGATCTTAACAAAGACGATCAAAAGTTGGGATCCTCATGAAGTGAGAAACCTCCCCGAAAATATCAAGTTATGCTATAAGATACTTTACAATTTTATTGAGGAGATTACCACATGTACTCTATTGGATCATGGGTGCAATGTTATGCCCTTTCTAAAAGAAGAGTGGGAGAATTTATGTGGAGCATTTTTGGTAGAAGCAAAATGGTTCCATGAAGGCTATACTCCTTCTCTTAAAGAATACCTAAAGAATGCGTGGATTTCAATTGGTGGGCCGATGACCTTTGTGTTTGCTTATTGTCTCCTAGGTCACACATTGGGAGATAATTCCCTTAATTGCTTAAAACAAGGTTTCGATCCTATATACTGGTCTTCACTGATACTCCGTCTCAATGATGATTTGGGAACTTCTAAGGTCGAAATGGAAAGAGGAGACACACCCAAATCAATTCAATGCCATATGAGAGAAACAGATGAATCAGATGAGGTATCTAGAGAATACATAAAGAACTTAGTGGACCATTTTTGGAAGGAGTTGAATCAAGAATCTATAAGAACTCATCTTCCAAAGAATTTTATGAACTTAGTCACGAACATGGCCCTGGCATCTCACTGCATCTTCCAATTTGGAGATGGAATTGGAGATTCCACTGGCATAACAAAAAATCGTATCCTCTCACTATTTTTCAATAATGTCCCATTGGAATGA

>LbdiTPS-2,MH203304

ATGGCAGCTATGAGCTGTCTCTCACTGGCCCGACTTCCCATCTCATCCACTTCTTCGAGTACTCGCAATTTCAGTTGTCTAACAAAACCTCATTCAATGCGGAGGTCATGCAAGATAGTCAATACTACGGAGAAATCGGAAGAACCTCCCCGTCGATCCGCCAATTACCACCCAACCATATGGGATCACTCGACCATCCAATCCATTGAGAATTTTAGTTCATTAAAGGTAATGTTCCAGGGGAACACACTTGAAAGGCGAAGAGAATCACTTAAGAATGAAGTGAAGTTATTGCTTGATGCATCAGACGATCCAGTGGCTCAGCTCCAGCTCATTGACACTATTCAACGGCTTGGCATCGCCTACCGTTTCGATAACGAGATTAAATCTATCCTTGATAGAATACGTGATTGTCATTTTGATCTAGAGGCTTTGGACTGTGTCCATAAGACGGCTCTTGCCTTTCGTCTACTCCGACAACATGGTTACGATGTGTCATCAGATGTTTTTCAGAAGTACAGAGACTCGCAAGGGTTCAAGGACTCCCTTACCGACGACGTGAAAGGACTGCTGAGCTTGTACGAAGCTTCTTTCCTTTCATTTCCAGGAGAACAACTGATGGAGGAAGCTAATAAGTTCAGCGTTAGGCACCTAGAGAGTTTGACTCAAAAGGTAGGGTTAGATATCGAGGAACAAGTGAGACACTCTCTACAAGTTCCATTGCATCGGAGGATGAGGAGACTCGAAGCTAGGGAGTACATAGATGTGTACCAGAGAGAGGGGGGGAAGAGCTCAGTCCTGCTAGAGTTTGCCAAGGTCGATTTCAACTTTGTGCAAATCATACATCAAATGGAATTGAAAGAACTCTCCAAATGGTGGATAAGCTTGAATTTGGGTAGTGTGCTCAGTTTTACCAGAGACAGATTAGTTGAAAATTATCTATGGGCTATCGGATTCGTCTATGAGCCCCATATGTCAAAATGTAGGATCGGCATCACTAAGGCGGTATGCATTTTATCCGTTATTGATGATGTCTATGATATATATGGGTCTTTTGAGGAAGTGGAGATCTTAACAAAGACGATCAAAAGTTGGGATCCTCATGAAGTGAGAAACCTCCCCGAAAATATCAAGTTATGCTATAAGATACTTTACAATTTTATTGAGGAGATTACCACATGTACTCTATTGGATCATGGGTGCAATGTTATGCCCTTTCTAAAAGAAGAGTGGGAGAATTTATGTGGAGCATTTTTGGTAGAAGCAAAATGGTTCCATGAAGGCTATACTCCTTCTCTTAAAGAATACCTAAAGAATGCGTGGATTTCAATTGGTGGGCCGATGACCTTTGTGTTTGCTTATTGTCTCCTAGGTCACACATTGGGAGATAATTCCCTTAATTGCTTAAAACAAGGTTTCGATCCTATATACTGGTCTTCACTGATACTCCGTCTCAATGATGATTTGGGAACTTCTAAGGTCGAAATGGAAAGAGGAGACACACCCAAATCAATTCAATGCCATATGAGAGAAACAGATGAATCAGATGAGGTATCTAGAGAATACATAAAGAACTTAGTGGACCATTTTTGGAAGGAGTTGAATCAAGAATCTATAAGAACTCATCTTCCAAAGAATTTTATGAACTTAGTCACGAACATGGCCCTGGCATCTCACTGCATCTTCCAATTTGGAGATGGAATTGGAGATTCCACTGGCATAACAAAAAATCGTATCCTCTCACTATTTTCCAATAATGTCCCATTGGAATGA

>LdiTPS-3,MH203298

ATGGCAGCTATGAGCTGTTTCTCACTGGCCCGACTTCCCATCTCATCCACTTCTTCGAGTACTCGCAATTTCAGTTGTCTAACAAAACCTCATTCAATTCAGAGGTCATGCAAGATAGTCAATACTACGGAGAAATTGGAAGAACCTCCCCGTCGATCCGCCAATTACCACCCAACCATATGGGATCACTCGACCATCCAATCCATTGAGAGTTTGAGTTCATTAAAGGGGAACACACCTGAAAGGCGAAGAGAATTACTTAAGAATGAAGTGAAGTTATTGCTTGATGCATCAGACGATCCAGTGGCTCAGCTCCAGCTCATTGACACTATTCAACGGCTTGGCATCGCCTACCATTTTAATAACGAGATTAAATCTATCCTTGATAGAATACATGATTGTCATGTTGATCTAGAGGCTTTGGACTGTGTCCATAAGACGGCTCTTGCCTTTCGTCTACTCCGACAACATGGTTACGATGTGTCATCAGATGTTTTTCAGAAGTACAGAGACTCGCAAGGGTTCAAGGACTCCCTTACCGACGACGTGAAAGGACTACTGAGCTTGTACGAAGCTTCTTTCCTTTCATTCCCAGGAGAACAACTGATGGAGGAAGCTAATAAGTTCAGCGTTAGGCACCTAGAGAGTTTGACTCAAAAGGTAGGGTTAGATATCGAGGAACAAGTGAGACGCTCTCTACAAGTTCCATTGCACCGGAGGATGAGGAGACTCGAAGCTAGGGAGTACGTAGATGTGTACCAGAGGGAGGAGGGGAAGAGCTCAGTCCTGCTAGAGTTTGCCAAGGTCGATTTCAACTTTGTGCAAATCATACATCAAATGGAATTGAAAGAACTCTCCAAGATCGGCATCACTAAGGCGGTATGCATTTTATCCGTTATTGATGATGTCTATGATATTTATGGGTCTTTTGAGGAAGTGGAGATCTTAACAAAGACGATCAAAAGTTGGGATCCTCATGAAATGAGAAACCTCCCCGAAAATATCAAGTTATGCTATAAGATAATTTACAATTTTATTGAGGAGATTACCACATCTATTCTATTGGATAATGGGTGCAATGTTATGCCCTTTCTAAAAAAAGAGTGGGAGAATTTATGTGGATCATATTTGGTAGAAGCAAAATGGTTCCATGAAGGCTATACTCCTTCTCTTAAAGAATACCTAAAGAATGCGTGGATTTCAATTGGTGGGCCGACGACCTTTGTTTTTGCTTATTGTCTCCTAGGACACACATTGGGAGATAATTCCCTTAATTGCTTAAAACAAGGTTTCGATCCTATATACTGGTCTTCACTAATACTCCGTCTCAATGATGATTTGGGAACTTCTAAGGCCGAAATGGAAAGAGGAGACACACCCAAATCAATTCAATGCCATATGAGAGAAACAGGTGAATCAGAAGAGGTATCTAGAGAATACATAAAGAACTTAGTGGACCATTTTTGGAAGGAGTTGAATCAAGAATCTATAAGAACTCATCTTCCGAAGAATTTTATGAACTTAGTCACGAACATGGCCCTAGCATCTCACTGCATTTTCCAATTTGGAGATGGAATTGGAGATTCCACTGGCATAACAAAAAATCGTATCCTCTCACTATTTGCCAATAATGTCCCATTGGAATGA

>LsoTPS-3,MH203275

ATGGCAGCTATGAGCTGTTTCTCACTGGCCCGACTTCCCATCTCATCCACTTCTTCGAGTACTCGCAATTTCAGTTGTCTAACAAAACCTCATTCAATTCAGAGGTCATGCAAGATAGTCAATACTACGGAGAAATTGGAAGAACCTCCCCGTCGATCCGCCAATTACCACCCAACCATATGGGATCACTCGACCATCCAATCCATTGAGAGTTTGAGTTCATTAAAGGGGAACACACTTGAAAGGCGAAGAGAATTACTTAAGAATGAAGTGAAGTTATTGCTTGATGCATCAGACGATCCAGTGGCTCAGCTCCAGCTCATTGACACTATTCAACGGCTTGGCATCGCCTACCATTTTAATAACGAGATTAAATCTATCCTTGATAGAATACATGATTGTCATGTTGATCTAGAGGCTTTGGACTGTGTCCATAAGACGGCTCTTGCCTTTCGTCTACTCCGACAACATGGTTACGATGTGTCATCAGATGTTTTTCAGAAGTACAGAGACTCGCAAGGGTTCAAGGACTCCCTTACCGACGACGTGAAAGGACTACTGAGCTTGTACGAAGCTTCTTTCCTTTCATTCCCAGGAGAACAACTGATGGAGGAAGCTAATAAGTTCAGCGTTAGGCACCTAGAGAGTTTGACTCAAAAGGTAGGGTTAGATATCGAGGAACAAGTGAGACACTCTCTACAAGTTCCATTGCACCGGAGGATGAGGAGACTCGAAGCTAGGGAGTACGTAGATGTGTACCAGAGGGAGGAGGGGAAGAGCTCAGTCCTGCTAGAGTTTGCCAAGGTCGATTTCAACTTTGTGCAAATCATACATCAAATGGAATTGAAAGAACTCTCCAAGATCGGCATCACTAAGGCGGTATGCATTTTATCCGTTATTGATGATGTCTATGATATTTATGGGTCTTTTGAGGAAGTGGAGATCTTAACAAAGACGATCAAAAGTTGGGATCCTCATGAAATGAGAAACCTCCCCGAAAATATCAAGTTATGCTATAAGATAATTTACAATTTTATTGAGGAGATTACCACATCTATTCTATTGGATAATGGGTGCAATGTTATGCCCTTTCTAAAAAAAGAGTGGGAGAATTTATGTGGATCATATTTGGTAGAAGCAAAATGGTTCCATGAAGGCTATACTCCTTCTCTTAAAGAATACCTAAAGAATGCGTGGATTTCAATTGGTGGGCCGACGACCTTTGTTTTTGCTTATTGTCTCCTAGGACACACATTGGGAGATAATTCCCTTAATTGCTTAAAACAAGGTTTCGATCCTATATACTGGTCTTCACTAATACTCCGTCTCAATGATGATTTGGGAACTTCTAAGGCCGAAATGGAAAGAGGAGACACACCCAAATCAATTCAATGCCATATGAGAGAAACAGGTGAATCAGAAGAGGTATCTAGAGAATACATAAAGAACTTAGTGGACCATTTTTGGAAGGAGTTGAATCAAGAATCTATAAGAACTCATCTTCCGAAGAATTTTATGAACTTAGTCACGAACATGGCCCTAGCATCTCACTGCATTTTCCAATTTGGAGATGGAATTGGAGATTCCACTGGCATAACAAAAAATCGTATCCTCTCACTATTTGCCAATAATGTCCCATTGGAATGA

>LrtTPS-3,MH203295

ATGGCAGCTATGAGCTGTTTCTCACTGGCCCGACTTCCCATCTCATCCACTTCTTCGAGTACTCGCAATTTCAGTTGTCTAGCAAAACCTCATTCAATTCAGAGGTCATGCAAGATAGTCAATACTACGGAGAAATTGGAAGAACCTCCCCGTCGATCCGCCAATTACCACCCAACCATATGGGATCACTCGACCATCCAATCCATTGAGAGTTTGAGTTCATTAAAGGGGAACACACTTGAAAGGCGAAGAGAATTACTTAAGAATGAAGTGAAGTTATTGCTTGATGCATCAGACGATCCAGTGGCTCAGCTCCAGCTCATTGACACTATTCAACGGCTTGGCATCGCCTACCATTTTAATAACGAGATTAAATCTATCCTTGATAGAATACATGATTGTCATGTTGATCTAGAGGCTTTGGACTGTGTCCATAAGACGGCTCTTGCCTTTCGTCTACTCCGACAACATGGTTACGATGTGTCATCAGATGTTTTTCAGAAGTACAGAGACTCGCAAGGGTTCAAGGACTCCCTTACCGACGACGTGAAAGGACTACTGAGCTTGTACGAAGCTTCTTTCCTTTCATTCCCAGGAGAACAACTGATGGAGGAAGCTAATAAGTTCAGCGTTAGGCACCTAGAGAGTTTGACTCAAAAGGTAGGGTTAGATATCGAGGAACAAGTGAGACACTCTCTACAAGTTCCATTGCACCGGAGGATGAGGAGACTCGAAGCTAGGGAGTACGTAGATGTGTACCAGAGGGAGGAGGGGAAGAGCTCAGTCCTGCTAGAGTTTGCCAAGGTCGATTTCAACTTTGTGCAAATCATACATCAAATGGAATTGAAAGAACTCTCCAAGATCGGCATCACTAAGGCGGTATGCATTTTATCCGTTATTGATGATGTCTATGATATTTATGGGTCTTTTGAGGAAGTGGAGATCTTAACAAAGACGATCAAAAGTTGGGATCCTCATGAAATGAGAAACCTCCCCGAAAATATCAAGTTATGCTATAAGATAATTTACAATTTTATTGAGGAGATTACCACATCTATTCTATTGGATAATGGGTGCAATGTTATGCCCTTTCTAAAAAAAGAGTGGGAGAATTTATGTGGATCATATTTGGTAGAAGCAAAATGGTTCCATGAAGGCTATACTCCTTCTCTTAAAGAATACCTAAAGAATGCGTGGATTTCAATTGGTGGGCCGACGACCTTTGTTTTTGCTTATTGTCTCCTAGGACACACATTGGGAGATAATTCCCTTAATTGCTTAAAACAAGGTTTCGATCCTATATACTGGTCTTCACTAATACTCCGTCTCAATGATGATTTGGGAACTTCTAAGGCCGAAATGGAAAGAGGAGACACACCCAAATCAATTCAATGCCATATGAGAGAAACAGGTGAATCAGAAGAGGTATCTAGAGAATACATAAAGAACTTAGTGGACCATTTTTGGAAGGAGTTGAATCAAGAATCTATAAGAACTCATCTTCCGAAGAATTTTATGAACTTAGTCACGAACATGGCCCTAGCATCTCACTGCATTTTCCAATTTGGAGATGGAATTGGAGATTCCACTGGCATAACAAAAAATCGTATCCTCTCACTATTTGCCAATAATGTCCCATTGGAATGA

>LapTPS-3,MH203296

ATGGCAGCTATGAGCTGTTTCTCGCTGGCCCGACTTCCCATCTCATCCACTTCTTCGAGTACTCGCAATTTCAGTTGTCTAACAAAACCTCATTCAATTCAGAGGTCATGCAAGATAGTCAATACTACGGAGAAATTGGAAGAACCTCCCCGTCGATCCGCCAATTACCACCCAACCATATGGGATCACTCGACCATCCAATCCATTGAGAGTTTGAGTTCATTAAAGGGGAACACACTTGAAAGGCGAAGAGAATTACTTAAGAATGAGGTGAAGTTATTGCTTGATGCATCAGACGATCCAGTGGCTCAGCTCCAGCTCATTGTCACTATTCAACGGCTTGGCATCGCCTACCATTTTAATAACGAGATTAAATCTATCCTTGATAGAATACATGATTGTCATGTTGATCTAGAGGCTTTGGACTGTGTCCATAAGACGGCTCTTGCCTTTCGTCTACTCCGACAACATGGTTACGATGTGTCATCAGATGTTTTTCAGAAGTACAGAGACTCGCAAGGGTTCAAGGACTCCCTTACCGACGACGTGAAAGGACTACTGAGCTTGTACGAAGCTTCTTTCCTTTCATTCCCAGGAGAACAACTGATGGAGGAAGCTAATAAGTTCAGCGTTAGGCACCTAGAGAGTTTGACTCAAAAGGTAGGGTTAGATATCGAGGAACAAGTGAGACACTCTCTACAAGTTCCATTGCACCGGAGGATGAGGAGACTCGAAGCTAGGGAGTACGTAGATGTGTACCAGAGGGAGGAGGGGAAGAGCTCAGTCCTGCTAGAGTTTGCCAAGGTCGATTTCAACTTTGTGCAAATCATGCATCAAATGGAATTGAAAGAACTCTCCAAGATCGGCATCACTAAGGCGGTATGCATTTTATCCGTTATTGATGATGTCTATGATATTTATGGGTCTTTTGAGGAAGTGGAGATCTTAACAAAGACGATCAAAAGTTGGGATCCTCATGAAATGAGAAACCTCCCCGAAAATATCAAGTTATGCTATAAGATAATTTACAATTTTATTGAGGAGATTACCACATCTATTCTATTGGATAATGGGTGCAATGTTATGCCCTTTCTAAAAAAAGAGTGGGAGAATTTTTGTGGATCATATTTGGTAGAAGCAAAATGGTTCCATGAAGGCTATACTCCTCCTCTTAAAGAATACCTAAAGAATGCGTGGATTTCAATTGGTGGGCCGACGACCTTTGTTTTTGCTTATTGTCTCCTAGGACACACATTGGGAGATAATTCCCTTAATTGCTTAAAACAAGGTTTCGATCCTATATACTGGTCTTCACTAATACTCCGTCTCAATGATGATTTGGGAACTTCTAAGGCCGAAATGGAAAGAGGAGACACACCCAAATCAATTCAATGCCATATGAGAGAAACAGGTGAATCAGAAGAGGTATCTAGAGAATACATAAAGAACTTAGTGGACCATTTTTGGAAGGAGTTGAATCAAGAATCTATAAGAACTCATCTTCCGAAGAATTTTATGAACTTAGTCACGAACATGGCCCTAGCATCTCACTGCATTTTCCAATTTGGAGATGGAATTGGAGATTCCACTGGCATAACAAAAAATCGTATCCTCTCACTATTTGCCAATAATGTCCCATTGGAATGA

>LamTPS-3,MH203284

ATGGCAGCTATGAGCTGTTTCTCACTGGCCCGACTTCCCATCTCATCCACTTCTTCGAGTACTCGCAATTTCAGTTGTCTAACAAAACCTCATTCAATTCAGAGGTCATGCAAGATAGTCAATACTACGGAGAAATTGGAAGAACCTCCCCGTCGATCCGCCAATTACCACCCAACCATATGGGATCACTCGACCATCCAATCCATTGAGAGTTTGAGTTCATTAAAGGGGAACACACTTGAAAGGCGAAGAGAATTACTTAAGAATGAAGTGAAGTTATTGCTTGATGCATCAGACGATCCATTGGCTCAGCTCCAGCTCATTGACACTATTCAACGGCTTGGCATCGCCTACCATTTCGATAACGAGATTAAATCTATCCTTGATAGAATACATGATTGTCATGTTCATCTAGAGGCTTTGGACTGTGTCCATAAGACGGCTCTTGCCTTTCGTCTACTCCGACAACATGGTTACGATGTGTCATCAGATGTTTTTCAGAAGTACAGAGACTCGCAAGGGTTCAAGGACTCCCTTACCGACGACGTGAAAGGACTGCTGAGCTTGTACGAAGCTTCTTTCCTTTCATTCCCAGGAGAACAACTGATGGAGGAAGCTAATAAGTTCAGCGTTAGGCACCTAGAGAGTTTGACTCAAAAGGTAGGGTTAGATATCGAGGAACAAGTGAGACACTCTCTACAAGTTCCATTGCACCGGAGGATGAGGAGACTCGAAGCTAGGGAGTACATAGATGTGTACCAGAGGGAGGAGGGGAAGAGCTCAGTCCTGCTAGAGTTTGCCAAGGTCGATTTCAACTTTGTGCAAATCATACATCAAATGGAATTGAAAGAACTCTCCAAGATCGGCATCACTAAGGCGGTATGCATTTTATCCGTTATTGATGATGTCTATGATATTTATGGGTCTTTTGAGGAAGTGGAGATCTTAACAAAGACGATCAAAAGTTGGGATCCTCATGAAATGAGAAACCTCCCCGAAAATATCAAGTTATGCTATAAGATAATTTACAATTTTATTGAGGAGATTACCACATCTATTCTATTGGATAATGGGTGCAATGTTATGCCCTTTCTAAAAAAAGAGTGGGAGAATTTATGTGGATCATATTTGGTAGAAGCAAAATGGTTCCATGAAGGCTATACTCCTTCTCTTAAAGAATACCTAAAGAATGCGTGGATTTCAATTGGTGGGCCGATGACCTTTGTTTTTGCTTATTGTCTCCTAGGACACACATTGGGAGATAATTCCCTTAATTGCTTAAAACAAGGTTTCGATCCTATATACTGGTCTTCACTAATACTCCGTCTCAATGATGATTTGGGAACTCCTAAGGCCGAAATGGAAAGAGGAGACACACCCAAATCAATTCAATGCCATATGAGAGAAACAGGTGAATCAGAAGAGGTATCTAGAGAATACATAAAGAACTTAGTGGACCATTTTTGGAAGGAGTTGAATCAAGAATCTATAAGAACTCATCTTCCGAAGAATTTTATGAACTTAGTCACGAACATGGCCCTAGCATCTCACTGCATTTTCCAATTTGGAGATGGAATTGGAGATTCCACTGGCATAACAAAAAATCGTATCCTCTCACTATTTGTCAATAATGTCCCATTGGAATGA

>LfoTPS-3,MH203286

ATGGCAGCTATGAGCTGTTTCTCACTGGCCCGACTTCCCATCTCATCCACTTCTTCGAGTACTCGCAATTTCAGTTGTCTAACAAAACCTCATTCAATTCAGGGGTCATGCAAGATAGTCAATACTACGGAGAAATTGGAAGAACCTCCCCGTCGATCCGCCAATTACCACCCAGCCATATGGGATCACTCGACCATCCAATCCATTGAGAGTTTGAGTTCATTAAAGGGGAACACACTTGAAAGGCGAAGAGAATTACATAAGAATGAAGTGAAGTTATTGCTTGATGCATCAGACGATCCAGTGGCTCAGCTCCAGCTCATTGACACTATTCAACGGCTTGGCATCGCCTACCATTTCGATAACGAGATTAAATCTATCCTTGATAGAATACATGATTGTCATGTTCATCTAGAGGCTTTGGACTGTGTCCATAAGACGGCTCTTGCCTTTCGTCTACTCCGACAACATGGTTACGATGTGTCATCAGATGTTTTTCAGAAGTACAGAGACTCGCAAGGGTTCAAGGACTCCCTTACCGACGACGTGAAAGGACTGCTGAGCTTGTACGAAGCTTCTTTCCTTTCATTCCCAGGAGAACAACTGATGGAGGAAGCTAATAAGTTCAGCGTTAGGCACCTAGAGAGTTTGACTCAAAAGGTAGGGTTAGATATCGAGGAACAAGTGAGACACTCTCTACAGGTTCCATTGCACCGGAGGATGAGGAGACTCGAAGCTAGGGAGTACATAGATGTGTACCAGGGGGAGGAGGGGAAGAGCTCAGTCCTGCTAGAGTTTGCCAAGGTCGATTTCAACTTTGTGCAAATCATACATCAAATGGAATTGAAAGAACTCTCCAAGATCGGCATCACTAAGGCGGTATGCATTTTATCCGTTATTGATGATGTCTATGATATTTATGGGTCTTTTGAGGAAGTGGAGATCTTAACAAAGACGATCAAAAGTTGGGATCCTCATGAAATGAGAAACCTCCCCGAAAATATCAAGTTATGCTATAAGATAATTTACAATCTTATTGAGGAGATTACCACATCTATTCTATTGGATAATGGGTGCAATGTTATGCCCTTTCTAAAAAAAGAGTGGGAGAATTTATGTGGATCATATTTGGTAGAAGCAAAATGGTTCCATGAAGGCTATACTCCTTCTCTTAAAGAATACCTAAAGAATGCGTGGATTTCAATTGGTGGGCCGATGACCTTTGTTTTTGCTTATTGTCTCCTAGGACACACATTGGGAGATAATTCCCTTAATTGCTTAAAACAAGGTTTCGATCCTATATACTGGTCTTCACTAATACTCCGTCTCAATGATGATTTGGGAACTTCTAAGGCCGAAATGGAAAGAGGAGACACACCCAAATCAATTCAATGCCATATGAGAGAAACAGGTGAATCAGAAGAGGTATCTAGAGAATACATAAAGAACTTAGTGGACCATTTTTGGAAGGAGTTGAATCAAGAATCTATAAGAACTCATCTTCCGAAGAATTTCATGAACTTAGTCACGAACATGGCCCTAGCATCTCACTGCATTTTCCAATTTGGAGATGGAATTGGAGATTCCACTGGCATAACAAAAAATCGTATCCTCTCACTATTTGTCAATAATGTCCCATTGGAATGA

>LbdrTPS-3,MH203280

ATGGCAGCTATGAGCTGTTTCTCACTGGCCCGACTTCCCATCTCATCCACTTCTTCGAGTACTCGCAATTTCAGTTGTCTAACAAAACCTCATTCAACTCAGAGGTCATGCAAGATAGTCAATACTACGGAGAAATTGGAAGAACCTCCCCGTCGATCCGCCAATTACCACCCAACCATATGGGATCACTCGACCATCCAATCCATTGAGAGTTTGAGTTCATTAAAGGGGAACACACTTGAAAGGCGAAGAGAATTACTTAAGAATGAAGTGAAGTTATTGCTTGATGCATCAGACGATCCAGTGGCTCAGCTCCAGCTCATTGACACTATTCAACGGCTTGGCATCGCCTACCATTTTAATAACGAGATTAAATCTATCCTTGATAGAATACATGATTGTCATGTTGATCTAGAGGCTTTGGACTGTGTCCATAAGACGGCTCTTGCCTTTCGTCTACTCCGACAACATGGTTACGATGTGTCATCAGATGTTTTTCAGAAGTACAGAGACTCGCAAGGATTCAAGGACTCCCTTACCGACGACGTGAAAGGACTACTGAGCTTGTACGAAGCTTCTTTCCTTTCATTCCCAGGAGAACAACTGATGGAGGAAGCTAATAAGTTCAGCGTTAGGCACCTAGAGAGTTTGACTCAAAAGGTAGGGTTAGATATCGAGGAACAAGTGAGACACTCTCTACAAGTTCCATTGCACCGGAGGATGAGGAGACTCGAAGCTAGGGAGTACGTAGATGTGTACCAGAGGGAGGAGGGGAAGAGCTCAGTCCTGCTAGAGTTTGCCAAGGTCGATTTCAACTTTGTGCAAATCATACATCAAATGGAATTGAAAGAACTCTCCAAGATCGGCATCACTAAGGCGGTATGCATTTTATCCGTTATTGATGATGTCTATGATATTTATGGGTCATTTGAGGAAGTGGAGATCTTAACAAAGACGATCAAAAGTTGGGATCCTCATGAAATGAGAAACCTCCCCGAAAATATCAAGTTATGCTATAAGATAATTTACAATTTTATTGAGGAGATTACCACATCTATTCTATTGGATAATGGGTGCAATGTTATGCCCTTTCTAAAAAAAGAGTGGGAGAATTTATGTGGATCATATTTGGTAGAAGCAAAATGGTTCCATGAAGGCTATACTCCTTCTCTTAAAGAATACCTAAAGAATGCGTGGATTTCAATTGGTGGGCCGACGACCTTTGTTTTTGCTTATTGTCTCCTAGGACACACATTGGGAGATAATTCCCTTAATTGCTTAAAACAAGGTTTCGATCCTATATACTGGTCTTCACTAATACTCCGTCTCAATGATGATTTGGGAACTTCTAAGGCCGAAATGGAAAGAGGAGACACACCCAAATCAATTCAATGCCATATGAGAGAAACAGGTGAATCAGAAGAGGTATCTAGAGAATACATAAAGAACTTAGTGGACCATTTTTGGAAGGAGTTGAATCAAGAATCTATAAGAACTCATCTTCCGAAGAATTTTATGAACTTAGTCACGAACATGGCCCTAGCATCTCACTGCATTTTCCAATTTGGAGATGGAATTGGAGATTCCACTGGCATAACAAAAAATCGTATCCACTCACTATTTGCCAATAATGTCCCATTGGAATGA

>LsoTPS-gDNA,MH618207

ATGGCAGCTATGAGCTGTTTCTCACTGGCCCAACTTCCCATCTTATTCACTTCTTCTTCAAGTACTCGCATTGTCAGTTGTCCAAGAAATCCTCGTTCAATTCAGAGGTCTTGCAAGATAGTCAATACTACTGAGAAAATGGAAGAACCTCCCCGTCGATCCGCCAATTACCACCCAACCATATGGGATCACTCGACCATCCAATCGATTCAGAGTTTGTGTTCATTAGAGGTAATGCTCTATGTAAGGCAGTTCCATGTAAGTGCTTTTGTTAGTTGGAAATCATTAAAACCCAATTACATTAAATAACAAGCATGCCCTCTCATTCTTGGCAGGGGAACACACTTGAAAGGCGGAGAGAATTACTTAAGATTGAAGTGAAGTTATTGCTTGATGCATCAGACGATCCATTGGCTCAGCTTCAACTCATTGACTCTATTCAACGGCTTGGCATCGCCTACCATTTCGATAACGAGATTAAATCTATCCTTGATAGAATATATGAATGTCATGTTGATCCAGAGGTATTGGGCTGTGTCGAAAAGACGGCTCTTGCCTTTCGTCTTCTCCGACAACATGGTTATGATGTATCTACAGGTAGTGTACGCACAGACCTCTAATGGCCCAAGATAAAAAGATATTTAGAAAAACATATTATCAAACAGTAATTGAGACATTTCTTAATACCAGTTGTTCCCGTACTCTTTGAAAAACATATTCTAAGATTTGCAGTGGATCTTTGTTTCTTCTAATTTCGGAGTGGTAGAACTCTAGTTTTTTAAAGTATTTGGTATTCTGAAAGAATTTTTCCATATTATTATATTGAACTTCGTAAATAAAACATATTCTAAATAGGGAGCTCTTCAATGTTAAAGAGAGATTCTTACCATGTTTTCGTGTTTGCTGCAGATGTTTTTCACAAGTACAGAAACTCACAAGGGTTCAAGGACTCCCTTACCCACGACGTGAAAGGAATGCTGAGCTTGTACGAGGCTTCTTTCCTTTCATTTCCAGGAGAGCAAGTGATGGAGGACGCTAAAGAGTCTAGCGTTAGGCATCTAGAGAATTTGACTCAAAATGTGAGATTGGATATCGAGGAACAAGTGAGACACTCTCTAGAAGTTCCATTGCACCGGAGGATGAGGAGACTCGAAGCTAGGGAGTACATAGATGTGTACCAAAGCAAAGAGGGGAAGAGCTGTGTCCTCCTGGAGTTTGCCAAGGTCGATTTCAACTATGTGCAAATTAGACATCAAGTGGAATTGAAAGAACTCTCCAAGTAAGATTACATTTTATGTAGCCTCTCGTAAATTTTTTTTTGTTTTCTTATTAGAAGCCCGTAATTTTTAAAATGTCAATAGAAATTTCGTTTTTCAAAATAAGAAATTTGTGGCTAGACAAGCTTTCCATGTTAATAGGTTAAGATATGGTCATTGAATTACTTATTAACCTTGTGAAATGAGTTAAGTTAATTAGTATTTCTATTTTAAAAGGTGGTGGACAAGCTTGGATTTGGAGAGTGTGCTCAGTTTCACAAGAGACAGATTAGTTGAGAATTATTTATGGGCTATCGGATTTGTCTATGAACCTCATATGTCAAAATGTAGGATCGGCCTCACTAAGGCGATATGCATTTTATCCATTATTGACGATGTCTATGATATTTATGGGTTTTTTGAGGAAGCGAAGATCTTAACAAAGACAATCAAAATGTAAGACAAGTTGATCATTGATCTACTTATTCTTATTTTCAAGTTATGTTGCTATCTTATAAAAAAATTGTATTTCTTTTTTCCAGATGGGATCCTCATGAAATGAGAAACCTCCCCAAAAATATCAAGTTATGTTATAAGATAATTCACAATTATATTGAGGAGATTACCACGGATATTTTATTGGATCATGGGTGCAATGTTATGCCAATTCTTAAAAAAGAGGTGGGATTAAATCTTCATAGATTTTCATTTCTATATTTTTTTTAAAAAAATAAATCATAATCTTGTTTTTACATATTCTAGTGGGAGATTTTTTGTGAAACACATTTGGTAGAAGCAAAATGGTTCAATGATGGGTATACTCCTTATCTTAAAGAATACATAGAGAATGCATGGATTTCAGCTGGTGGACCGATGGCCATGGTTTTTGCATATTGTCTCCAAGGTCACACTTTGGGAGATAATTCACTTAATTGCTTAAAACAAGGTTTCATTCCTATATATTGGTCTTCACTAATACTTCGTCTCAATGATGATTTGGCAACTTCTAAGGTAACAACTATTAACTCTTGTCTCTCAAATTTTCTCTGTAACTATCCTACTAAACTATAACATATTATTGTTCCTAATATTTTTTTAACTCTTTTGAAAGGCCGAGATGGAAAGAGGGGACAATGACAAATCAATTCAGTGCTATATGAGAGAAACAAATGAACCAGAAGATGTATCAAGAGAATACATACAAAACTTAGTGGACCATTCTTGGAAGGAGTTGAATCAAGAATATATAAGAACTCATCTTCCAAAGAATTTTATGAACGTAGTCGTGAACATGGCCAGAGCGTCTCACTGTATTTTCCAATATGGAGACGGAATTGGAGATTCCACTGGCACAACAAAAAATCATATCCTCTCTCTATTTTTCAATAATGTCCCATTGGAATGA
